# Ge-Gen-Qin-Lian decoction alleviates the symptoms of type 2 diabetes mellitus with inflammatory bowel disease via regulating the AGE-RAGE pathway

**DOI:** 10.1186/s12906-024-04526-x

**Published:** 2024-06-10

**Authors:** Zhipeng Li, Ziwei Zhao, Shujuan Chen, Xiaojuan Wang, Dongsheng Wang, Xiaoli Nie, Ye Yao

**Affiliations:** 1 Department of Nephrology, Southern Medical University Hospital of Integrated Traditional Chinese and Western Medicine, Southern Medical University, No. 13, Shi Liu Gang Road, Haizhu District, Guangzhou, Guangdong Province 510315 China; 2 Institute of Integrated Traditional Chinese and Western Medicine, Xiangya Hospital, Central South University, Changsha, 410008 China

**Keywords:** Ge-Gen-Qin-Lian decoction, Type 2 diabetes mellitus, Inflammatory bowel disease, AGE-RAGE pathway, Network pharmacology

## Abstract

**Background:**

This study aimed to explore the mechanism of Ge-Gen-Qin-Lian decoction (GGQLD) in the alleviation of symptoms of type 2 diabetes mellitus (T2DM) with inflammatory bowel disease (IBD) by network pharmacology and experimental validation.

**Methods:**

The active components and targets of GGQLD were identified from the TCMSP database. The potential therapeutic targets of T2DM and IBD were identified from the GEO database and 4 online disease target databases. The PPI network and KEGG/GO analyses were performed with the common targets among GGQLD, T2DM and IBD. Molecular docking was carried out between the core compounds and hub targets. To verify the above results, UHPLC-MS technology was used to identify the chemical compounds in GGQLD, and a T2DM with IBD rat model was used to explore the mechanism by which GGQLD treats T2DM with IBD.

**Results:**

Totally, 70 potential therapeutic targets were identified among GGQLD, T2DM and IBD. Ten hub genes were selected from the PPI network. KEGG analysis revealed that GGQLD is tightly involved in the AGE-RAGE signaling pathway. Berberine, baicalein, wogonin, and quercitrin are the main active compounds of GGQLD. Animal experiments showed that GGQLD could decrease blood glucose and alleviate intestinal inflammation. Notably, the concentrations of AGEs, the expression of RAGE, c-JUN and NF-κB and the expression of inflammatory cytokines were decreased by GGQLD.

**Conclusions:**

Our study initially demonstrated that GGQLD has favorable anti-hyperglycemic and anti-intestinal inflammation effects in a T2DM with IBD rat model, and the AGE-RAGE pathway plays a vital role in this process.

**Supplementary Information:**

The online version contains supplementary material available at 10.1186/s12906-024-04526-x.

## Introduction

Type 2 diabetes mellitus (T2DM) is one of the most common chronic diseases, and approximately 1 in 10 adults worldwide are estimated to have T2DM [1]. T2DM and its life-threatening complications lead to reduced quality of life, increased mortality and increased healthcare costs [2]. High-fat and high-sugar diets, decreased amounts of physical exercise, dysregulation of the intestinal microenvironment and genetic susceptibility contribute to the development of T2DM [3, 4]. Inflammatory bowel disease (IBD), which includes ulcerative colitis (UC) and Crohn's disease (CD), is a chronic inflammatory disease involving the ileum, rectum and colon. Interestingly, the above risk factors related to T2DM also contribute to the progression of IBD [5, 6]. Thus, T2DM and IBD seem to be closely related.

Intestinal barrier injury and intestinal inflammation are the mechanisms underlying both T2DM and IBD [7]. The intake of high-fat and high-sugar diets disrupts the composition of gut microbes and directly or indirectly destroys intestinal barrier integrity [5, 8]. Lipopolysaccharides, gut microbes, and metabolites, which are present in the intestine, cross the damaged intestinal barrier and trigger systemic inflammatory reactions, resulting in the production of proinflammatory cytokines [8, 9]. Proinflammatory cytokines, including tumor necrosis factor-α (TNF-α), interleukin 6 (IL-6), and interleukin 1β (IL-1β), have been shown to suppress insulin signal transduction and have crucial deleterious effects on the development of IBD [10–14].

Furthermore, hyperglycemia and hyperlipidemia induce the accumulation of advanced glycation end products (AGEs) [15]. The receptor for advanced glycation end products (RAGE) can be activated by AGEs, subsequently triggering the activation of many inflammatory signaling pathways [15, 16]. Many studies have shown that the downstream consequences of the AGE-RAGE axis involve compromised insulin signaling, perturbation of metabolic homeostasis, and disruption of intestinal barrier function [17]. Additionally, a study revealed that improving intestinal mucosal barrier function through the regulation of AGE-RAGE signaling could improve recovery in UC patient [18]. Another study demonstrated that the TT genotype and the A allele of RAGE-374T/A polymorphisms were related to CD and UC risk [19]. Inhibition of the AGE-RAGE signaling pathway could alleviate intestinal inflammation and restore the intestinal mucosa barrier, which might be the common pathological mechanism of T2DM and IBD [7].

Ge-Gen-Qin-Lian decoction (GGQLD) is one of the most well-known traditional Chinese medicinal formulas and comprises 4 Chinese herbs: *Puerariae Lobatae* Radix (Gegen), *Scutellaria baicalensis* Georgi. (Huangqin), *Coptis chinensis* Franch. (Huanglian) and *Glycyrrhiza uralensis* Fisch. (Gancao).It was first recorded in “Shang Han Lun” to treat diarrhea, dysentery and malaria by Zhongjing Zhang during the Han Dynasty. “Syndrome differentiation” is the basic rule of traditional Chinese medicine (TCM) for treating disease, which refers to which therapeutic schedule will be chosen based on the syndrome but not the disease. According to TCM theory, intestinal damp heat is the most common traditional Chinese medical pathogenesis for not only intestinal disease but also T2DM (called “Xiaoke” in TCM). GGQLD has the ability to eliminate intestinal damp heat. Therefore, GGQLD treating T2DM with IBD is supported by TCM theory.

Many recent in vivo and in vitro studies have also shown that GGQLD can treat T2DM and IBD well. A previous animal study revealed that GGQLD could protect against UC (belonging to the IBD group) by ameliorating inflammation and downregulating the EGFR/PI3K/AKT signaling pathway [20]. Moreover, animal experiments revealed that GGQLD could alleviate chemotherapy-induced IBD [21, 22]. Our previous study found that in a T2DM rat model compared with chronic bowl inflammatory response, and berberine, the most important major active ingredient of GGQLD, could alleviate T2DM by regulating gut microbes and intestinal mucosal barrier dysfunction [23, 24]. Furthermore, an in vitro study revealed that the antidiabetic nanoaggregates from GGQLD increased the absorption of baicalin and cellular antioxidant activity. Additionally, some in vivo studies have shown that GGQLD can alleviate the symptoms of T2DM [25–27]. The effects of GGQLD on T2DM have been supported by Chinese medical specialists, and the use of GGQLD is described in the Guidelines for the Prevention and Treatment of Type 2 Diabetes Mellitus in China [28]. The therapeutic effects of GGQLD on T2DM and IBD have been repeatedly proven by in vitro and in vivo studies, but the underlying mechanisms of GGQLD are unclear.

**Fig. 1:**
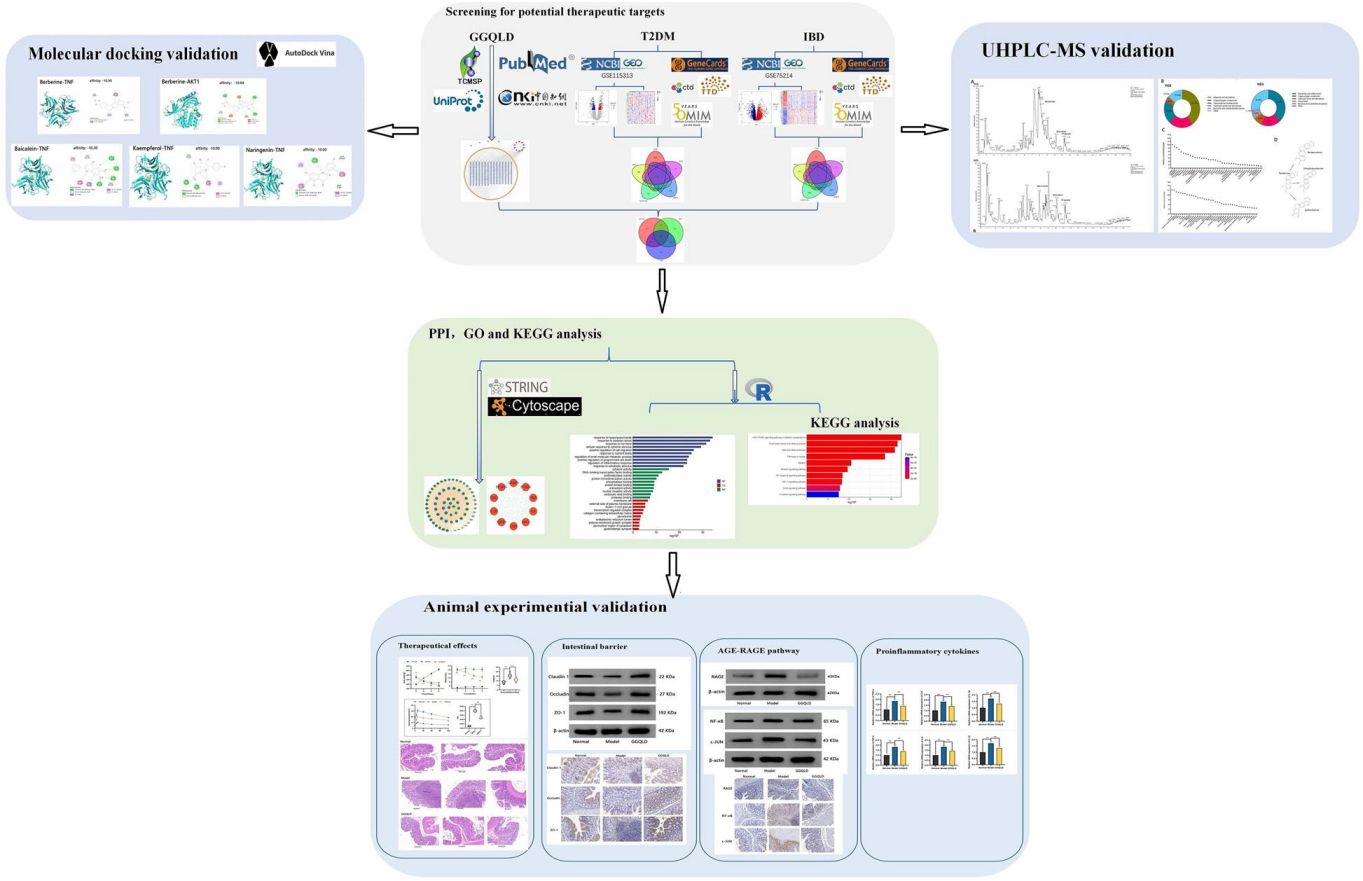
Flowchart illustrating the study of GGQLD for treating T2DM with IBD

In this study, network pharmacology, bioinformatics and molecular docking approaches were applied to identify the key compounds and potential mechanisms of GGQLD in treating T2DM patients with IBD. Finally, we verified the chemical compounds of GGQLD via ultrahigh-performance liquid chromatography‒mass spectrometry (UHPLC-MS), and the mechanism by which GGQLD treats T2DM with IBD was proven in a rat model via molecular biology experiments (Fig. 1).

## Materials and methods

### Network pharmacology analysis

#### Screening for the active components and targets of GGQLD

The Traditional Chinese Medicine Systems Pharmacology Database and Analysis Platform (TCMSP) tool (https://tcmsp-e.com/) was used to identify the active components of GGQLD. There are 4 herbs, namely, Gegen (GG), Huanglian (HL), Huangqin (HQ) and Gancao (GC), in GGQLD. We obtained the selected active components by searching for the following criteria: oral bioavailability (OB) ≥ 30% and drug likeness (DL) ≥ 0.18. The protein targets of these selected active components were also searched in TCMSP, and the IDs were converted to gene symbols with the UniProt database (http://www.uniprot.org). The chemical components and targets were obtained from published literature in the China National Knowledge Infrastructure (CNKI) and PubMed repositories. Finally, Cytoscape 3.9.1 was used to construct the herb–component–target network, and betweenness indices were calculated to assess the core compounds of GGQLD.

#### Screening for GGQLD-T2DM-IBD-related targets

The data in the gene microarray dataset GSE115313 were obtained from the colonic tissue of T2DM patients, and those in the GSE75214 dataset were obtained from the colonic tissue of IBD patients (http://www.ncbi. nlm.nih.gov/geo/). Data from both datasets were obtained from the National Center for Biotechnology Information (NCBI) repository. The DEGs between healthy controls and patients were identified using the GEO2R online tool. We set a *p* value of < 0.05 as the cutoff criterion. The R packages ggplot2 and pheatmap were used to visualize the DEGs on volcano plots and heatmaps, respectively.

Furthermore, potential therapeutic targets in T2DM and IBD were mined from the GeneCards database (http://www.genecards.org/), the Comparative Toxicogenomics Database (CTD) (https://ctdbase.org/), the Online Mendelian Inheritance in Man (OMIM) database (https://omim.org/) and the Therapeutic Target Database (TTD) (https://db.idrblab.net/ttd/) through a search with the key words “type 2 diabetic mellitus” and “inflammatory bowel disease”.

The intersection between the DEGs and the therapeutic targets mined from each database was determined by constructing a Venn diagram, and the targets that appeared at least 2 times in each database were considered therapeutic T2DM-IBD targets. Finally, the T2DM-IBD targets were intersected with the GGQLD targets, and the overlapping targets were selected as the targets of interest for further analysis.

#### Construction of a protein‒protein interaction (PPI) network

A total of 70 targets of interest for the regulatory effects of GGQLD on T2DM and IBD were identified. All 70 targets of interest were imported into the Search Tool for the Retrieval of Interacting Genes/Proteins (STRING) tool (version: 11.5) (https://cn.string-db.org/), after which a PPI network of GGQLD was constructed. Then, we assessed the topological properties of every node in the interaction network by calculating three parameters with the Cytoscape plugin CytoNCA: degree, betweenness centrality (BC) and closeness centrality (CC). The targets with the 10 highest degree values were considered the hub targets.

#### Kyoto Encyclopedia of Genes and Genomes (KEGG) pathway and Gene Ontology (GO) term enrichment analyses

The 70 targets of interest were imported into Metascape online software (https://metascape.org/) for KEGG and GO enrichment analyses. For GO analysis, three categories were considered: biological process (BP), molecular function (MF) and cellular component (CC). Significant enrichment was defined by a *p* value of ≤ 0.01, and the top 10 KEGG pathways and GO pathways were visualized with the ggplot2 R package.

#### Molecular docking validation

The 6 core compounds of GGQLD and 10 hub targets were used for molecular docking validation via AutoDock Vina (version: 1.2.3) (https://vina.scripps.edu/). KEGG pathway and GO term enrichment analyses revealed that the AGE-RAGE signaling pathway in diabetic complications was the most important pathway involved in the therapeutic effect of GGQLD on T2DM with IBD. However, RAGE was not included among the targets of GGQLD because RAGE data were not stored in the TCMSP. Hence, we explored the effects of GGQLD on RAGE, and the interactions between the 6 core compounds of GGQLD and RAGE were validated via molecular docking analysis.

### Experimental validation

#### Main reagents

The details of the main reagents used are provided in File S1.

#### Preparation of GGQLD

GGQLD consists of 4 Chinese herbs, i.e., *Puerariae Lobatae* Radix (Gegen), *Scutellaria baicalensis* Georgi. (Huangqin), *Coptis chinensis* Franch. (Huanglian) and *Glycyrrhiza uralensis* Fisch. (Gancao), at a ratio of 8:3:3:2 (Table 1). All the herbs in GGQLD were purchased from Xiangya Hospital, Central South University, and were authenticated by the pharmacist Xinzhong Li. The extracts of GGQLD were prepared according to previous methods [26, 27, 29]. In brief, a total of 3200 g of GGQLD was immersed in a tenfold volume of distilled water (w/v) for 0.5 h, and the compounds were then extracted by two rounds of reflux extraction (40 min and 30 min). Later, the reflux condensate was filtered to remove the herbal residue. The two filtrates were combined and evaporated to dryness. Before the animal experiment and UHPLC-MS analysis, we added distilled water to dissolve the GGQLD powder and ensured that the concentration of GGQLD was 2 g/mL (g of crude drug per mL) for further studies[29].

**Table 1:** Herbal composition of GGQLD

Latin plant name	Chinese name	Family	Used part	Proportion
*Puerariae Lobatae* Radix	Gegen	*Leguminosae*	Root	8
*Scutellaria baicalensis* Georgi	Huangqin	*Lamiaceae*	Root	3
*Coptis chinensis* Franch	Huanglian	*Ranunculaceae*	Root	3
*Glycyrrhiza uralensis* Fisch	Gancao	*Leguminosae*	Root	2

#### Identification of chemical compounds in GGQLD

UHPLC-MS was used for relative quantification of the chemical compounds in GGQLD. The details are provided in File S1.

#### Animal experiment

A total of 30 male specific pathogen-free (SPF) Sprague‒Dawley (SD) rats (6 weeks old, weighing 220 ± 20 g) were purchased from the Department of Laboratory Animals, Central South University (Changsha, China; permission code: SCXK2019-0004, batch number: 430727231103690366). The rats were raised in groups of 3 rats per cage in a room with a relative humidity of 50 ± 15%, a temperature of 25 ± 2°C, and a 12-h dark–light cycle. The rats were provided sterile water and a sterile diet. This animal experiment was approved by the Institutional Animal Care and Use Committee of Central South University (Changsha, China; permit number: CSU-2022–0063).

After 1 week of acclimatization, the 30 rats were randomly divided into 3 groups (10 rats per group): the normal group, the model group and the GGQLD group. Rats in the model and GGQLD groups were fed a high-fat diet, and rats in the normal group were fed a normal diet. The compounds of the high-fat diet used were described in our previous studies. After 6 weeks, the rats in the model and GGQLD groups were injected intraperitoneally with streptozotocin (STZ, 35 mg/kg) [30]. Later, the rats in the above 2 groups were given water containing 3% dextran sodium sulfate (DSS) for 7 days [31]. Rats with a fasting blood glucose (FBG) concentration of ≥ 16.7 mol/L and hematochezia were considered diabetic and to have intestinal inflammation.

The rats in the GGQLD group were treated daily with GGQLD (20 g of crude drug/kg/day or 10 mL of GGQLD herbal liquid/kg/day) via intragastric administration. The rats in the normal and model groups were given an equal volume of distilled water via intragastric administration. The interventions were continued for 6 weeks. The rats were anesthetized via isoflurane inhalation (concentration maintained at approximately 2%, Shanghai Abbott Laboratories, Shanghai, China), the chest was opened, and the rats were sacrificed after blood collection through cardiac puncture. The blood was centrifuged at 3500 rpm (4°C) for 15 min to obtain the serum. The colon was rinsed with phosphate-buffered saline. A portion of the colon was stored in 4% polyformaldehyde solution (in the dark, 4°C) for hematoxylin and eosin (H&E) staining and immunohistochemistry, and another portion of the colon was stored at -80°C for Western blot and quantitative real-time PCR (qRT‒PCR) analyses. The design of the animal experiment is shown in Fig. 7A.

#### Biochemical assays

FBG was measured with a glucometer (Roche Diagnostics GmbH, Germany) via tail vein sampling after 8 h of fasting. An oral glucose tolerance test (OGTT) was performed after 6 weeks of treatment, and the steps described in our previous studies were followed [23]. The area under the curve (AUC) was calculated using the following formula: 0* BG + 5*BG + 10*BG + 30*BG + 60*BG + 120*BG (BG, blood glucose). The concentration of insulin was measured with an ELISA kit (SEKM-0141, Solarbio, Beijing, China). The homeostatic model assessment for insulin resistance (HOMA-IR) index was calculated as follows: FBG (mmol/l) * insulin concentration (mU/l) ⁄22.5.

The concentrations of AGEs were measured by an ELISA kit (ab273298, Abcam, Cambridge, UK). Three cytokines (IL-6, IL-1β and TNF-α) were also quantified by ELISA kits (E-EL-R0015, E-EL-R0016 and E-EL-R0011, Elabscience, Wuhan, China), and all the procedures were performed in accordance with the manufacturer’s instructions.

#### H&E staining

Colonic tissues were fixed with 4% polyformaldehyde solution, dewaxed and then stained with H&E staining solution (B-SMS250, Chande BKMAM Biotechnology Co., Ltd., Changde, China). Following the dehydration and clearing steps, the sections were mounted with neutral gum for imaging (*n* = 3 rats/group, Pannoramic SCAN, 3DHISTECH, Budapest, Hungary).

#### Immunohistochemical staining

Immunohistochemical staining was used to analyze the expression of Claudin-1, Occludin, ZO-1, RAGE, NF-κB, and c-JUN. The steps used were as follows: paraffin sectioning, dewaxing in water, antigen retrieval, blocking of endogenous peroxidase activity, addition of the corresponding antibodies, nuclear staining, and mounting. Antigen retrieval was performed with citric acid (pH 6.0) in a microwave at medium power for 8 min, zero power for 8 min, and medium–low power for 7 min. The nuclei of the hematoxylin-stained sections appeared blue, and positive reactions with antibodies were indicated by brownish-yellow staining. Images were captured using a pathological section scanner, and the mean integrated optical density (IOD) was calculated using Image‑Pro Plus 6.0 software and used to quantify staining.

#### Western blot analysis

Rat colonic tissues were lysed with a commercial kit (P0028, Beyotime Biotechnology Co. Ltd., Shanghai, China), and protein concentrations were quantified according to the manufacturer’s instructions. Equal amounts of protein were separated on sodium dodecyl sulfate–polyacrylamide gel electrophoresis (SDS‒PAGE) gels (Bio-Rad) and transferred to polyvinylidene fluoride (PVDF) membranes (0.22μm; Biosharp, Anhui, China). The membranes were then blocked with 5% nonfat milk and incubated with antibodies against claudin-1, occludin, ZO-1, RAGE, NF-κB, c-JUN and β-actin overnight (AWA41881, AWA49018, AWA47635, AWA10402, AWA01427 and AWA10453, Abiowell, Changsha, China). After incubation with the corresponding secondary antibodies for 2 hours, the target proteins were visualized using a multifunctional imaging system (Shenhua Science Technology, Hangzhou, China).

#### qRT‒PCR

Total RNA from colonic tissues was isolated using TRIzol (Invitrogen Life Technologies Co., Ltd., California, USA), and cDNA was synthesized with a RevertAid First Strand cDNA Synthesis Kit (Thermo Fisher Scientific, Massachusetts, USA). qRT‒PCR was performed using a CFX Connect system (Bio-Rad, USA) with MonAmp™ SYBR® Green qPCR Mix. The primers for claudin-1, occludin, ZO-1, RAGE, NF-κB, c-JUN, TNF-α, IL-1βand IL-6 were synthesized by Sangon Biotech Technology Co., Ltd., and are listed in FileS1.

### Statistical analysis

The data are reported as the means ± standard deviations (SDs), and analysis was conducted using SPSS 23.0. GraphPad Prism 9.0 software was used for graphical presentation. The heatmaps, Venn diagrams and volcano plots were generated with R software. Student’s t test was performed to compare data between two groups. *p* < 0.05 was considered to indicate statistical significance.

## Results

### The active components and targets of GGQLD

**Fig. 2:**
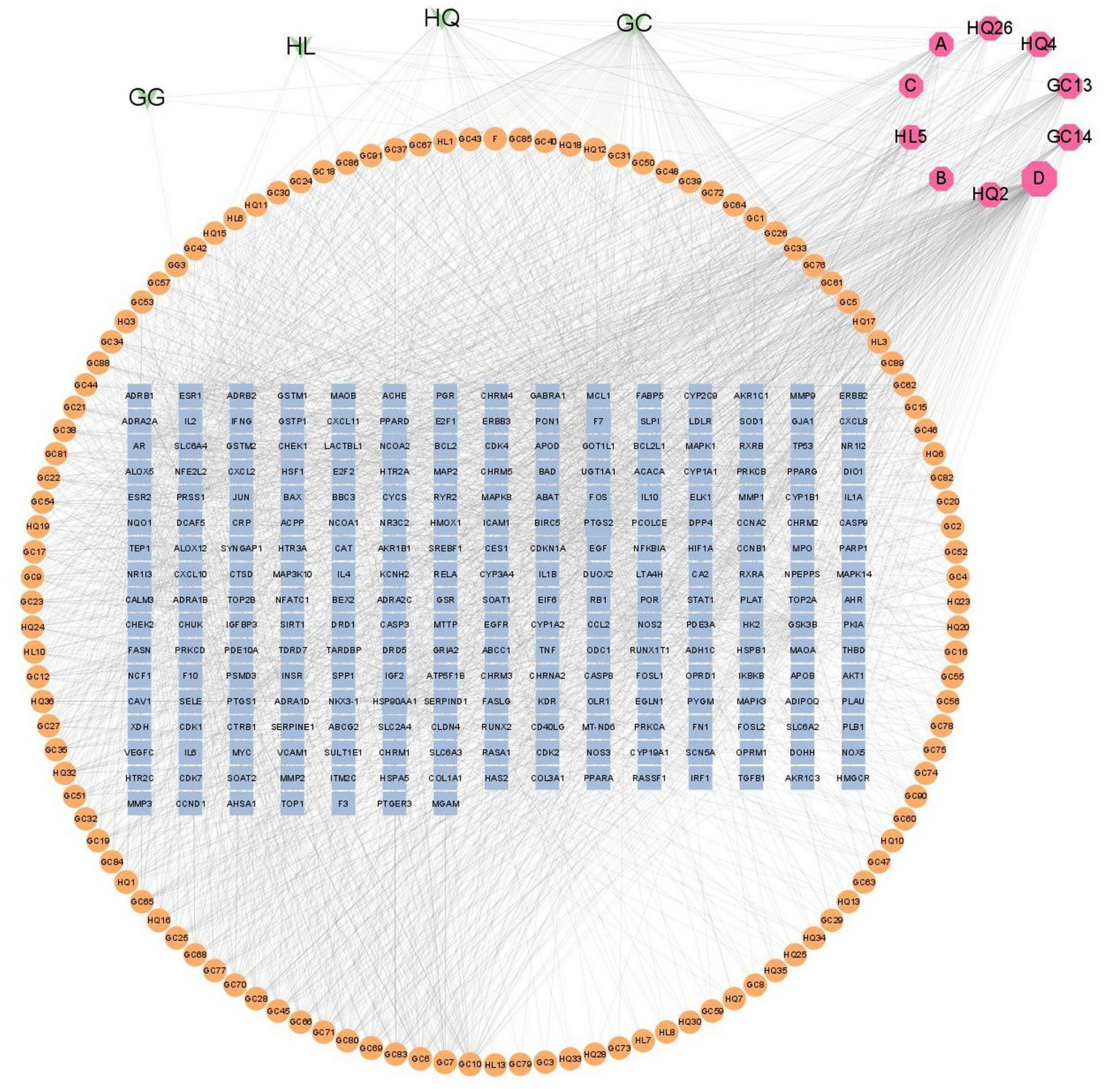
Herb-component-target network of GGQLD

Based on the TCMSP database, 146 active components of GGQLD were obtained, namely, 4 from Gegen, 14 from Huanglian, 36 from Huangqin and 92 from Gancao, with 6 components overlapping components (Table S1). In total, 266 targets of the 146 components were identified (File S2), and an herb–component–target network was constructed with Cytoscape 3.9.1. The betweenness indices were calculated, and the 5 most important active compounds with the highest betweenness indices were quercetin, naringenin, kaempferol, baicalein and wogonin (Fig. 2). Many studies have reported that berberine (an active component of Huanglian) can be used to treat T2DM and IBD [32–34]. Thus, berberine was deemed a core ingredient and is listed in Table 2.

**Table 2:** The 6 core compounds of GGQLD

ID	Mol ID	Molecule Name	Molecular Weight	Structure	OB (%)	DL	Betweenness	Source
D	MOL000098	quercetin	302.25		46.43	0.28	60,170.11	Huanglian& Gancao
GC14	MOL004328	naringenin	272.27		59.29	0.21	15,143.761	Gancao
GC13	MOL000422	kaempferol	286.25		41.88	0.24	11,053.099	Gancao
HQ4	MOL002714	baicalein	270.25		33.52	0.21	8897.182	Huangqin
HQ2	MOL000173	wogonin	284.28		30.68	0.23	7080.946	Huangqin
HL1	MOL001454	berberine	336.39		36.86	0.78	611.233	Huanglian

### Identification of the targets for GGQLD in the treatment of T2DM and IBD

**Fig. 3:**
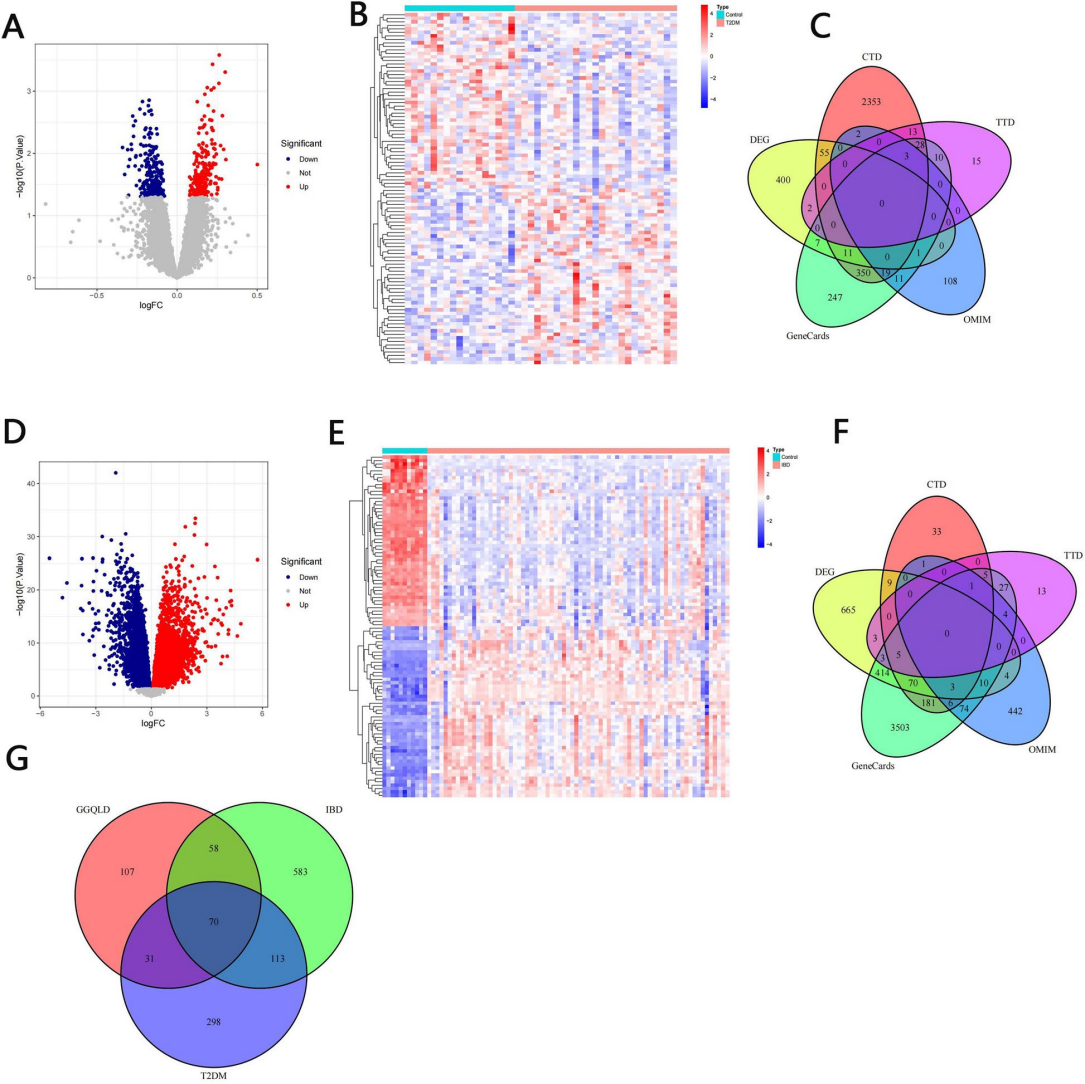
Identification of targets associated with GGQLD, T2DM and IBD. Volcano plot (**A**) and heatmap (**B**) of the DEGs in the colon between T2DM samples and healthy control samples. **C** Venn diagram of the T2DM targets identified in 4 disease databases and the DEGs. Volcano plot (**D**) and heatmap (**E**) of the DEGs in the colon between IBD samples and healthy control samples. **F** Venn diagram of the IBD targets identified in 4 disease databases and the DEGs. **G** Venn diagram of the T2DM targets, IBD targets and GGQLD targets

The GSE115313 dataset contains 17 samples from healthy controls and 25 samples from patients with T2DM. In total, 477 DEGs in colonic tissue between the healthy control and T2DM samples were identified based on the selection criteria; 247 DEGs were upregulated and 230 were downregulated (Fig. 3A, B). Furthermore, 687 T2DM targets were obtained from the GeneCards database (relevance score ≥ 5), 144 T2DM targets were obtained from the OMIM database, 73 T2DM targets were obtained from the TTD, and 2834 T2DM targets were obtained from the CTD (Fig. 3C). The T2DM targets appearing in at least two of the above databases were selected as T2DM targets. Finally, 512 T2DM targets were identified in this study (File S3).

The GSE75214 dataset contains 11 samples from healthy controls and 74 samples from patients with IBD. A total of 11,223 DEGs were identified in this dataset. However, only 1186 DEGs satisfied the filtering criteria: 755 upregulated DEGs and 431 downregulated DEGs (Fig. 3D, E). Furthermore, 4306, 547, 62 and 361 IBD targets were identified in the GeneCards database (relevance score ≥ 5), OMIM database, TTD, and CTD, respectively (Fig. 3F, File S3). A total of 824 IBD targets appeared in at least two of the above databases.

Overall, 70 potential therapeutic targets overlapped among the GGQLD, T2DM and IBD patients, and these targets were considered the targets of interest for further analysis (Fig. 3G).

### PPI network analysis, KEGG pathway analysis and GO pathway analysis

**Fig. 4:**
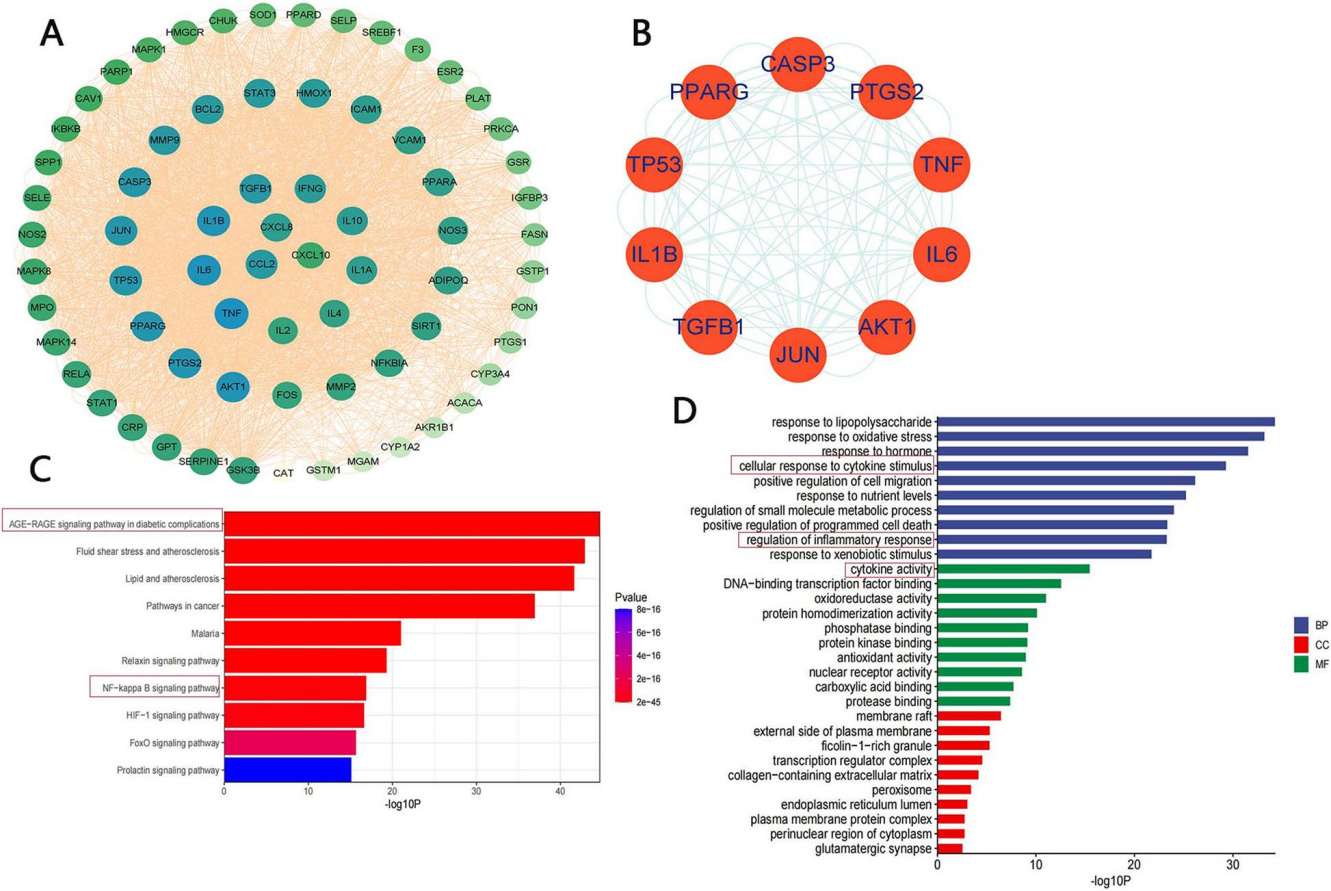
Results of the PPI network analysis, KEGG pathway enrichment analysis and GO term enrichment analysis for the 70 potential therapeutic targets. **A** PPI network of the 70 potential therapeutic targets. **B** PPI network of the 10 hub targets. **C** Results of KEGG pathway enrichment analysis. **D** Results of GO term enrichment analysis; BP: biological process, MF: molecular function, CC: cellular component

A PPI network containing 70 nodes and 2809 edges was constructed with STRING online software (Fig. 4A). The larger and bluer the circle, the greater the influence of the target on the disease. The core targets comprised nine cytokine genes (TNF, IL-6, IL-1B, TGF-B1, IFNG, IL-10, IL-1A, IL-4 and IL2) and 3 chemokine genes (CCL2, CXCL8 and CXCL10), indicating that the mechanisms by which GGQLD improves the symptoms of T2DM with IBD are closely related to the inflammatory response (Fig. 4A). The top 10 targets of interest, namely, TNF, IL6, AKT1, IL1B, PTGS2, PPARG, TP53, JUN, CASP3, and TGFB1, were selected as the hub targets (Fig. 4B, Table 3). To explore the mechanism of GGQLD in treating T2DM compared with IBD, we performed molecular docking validation to identify the docking interactions between the 6 core compounds and 10 hub targets.

**Table 3:** The top 10 degrees in PPI with 70 potential therapeutic targets

Target	Degree	Betweenness	Closeness	Target	Degree	Betweenness	Closeness
TNF	134	152.12	0.97	PPARG	122	93.29	0.90
IL6	130	124.95	0.95	TP53	120	100.68	0.88
AKT1	128	108.72	0.93	JUN	118	69.27	0.87
IL1B	126	88.79	0.92	CASP3	118	56.14	0.87
PTGS2	124	79.98	0.91	TGFB1	116	56.26	0.85

KEGG pathway enrichment analysis revealed that AGE-RAGE signaling pathway in diabetic complications was the most important pathway (Fig. 4C, Table 4). Many studies have shown that high glucose conditions induce the production of AGEs and that AGEs can immediately activate the RAGE signaling pathway [15–17]. Then, the NF-κB/c-JUN signaling pathway is activated, and large amounts of inflammatory cytokines are released to elicit an inflammatory response [17]. The results of GO term enrichment analysis also revealed that the mechanism of GGQLD in treating T2DM compared with IBD is related to the inflammatory response (Fig. 4D).

**Table 4:** The top 10 KEGG pathways with 70 potential therapeutic targets

KEGG ID	Description	Count	%	*P*value	Gene symbol
hsa04933	AGE-RAGE signaling pathway in diabetic complications	25	35.71	2.039E-45	AKT1, BCL2, CASP3, MAPK14, F3, ICAM1, IL1A, IL1B, IL6, CXCL8, JUN, MMP2, NOS3, SERPINE1, PRKCA, MAPK1, MAPK8, RELA, CCL2, SELE, STAT1, STAT3, TGFB1, TNF, VCAM1
hsa05418	Fluid shear stress and atherosclerosis	26	37.14	1.269E-43	AKT1, BCL2, CAV1, CHUK, MAPK14, FOS, GSTM1, GSTP1, HMOX1, ICAM1, IFNG, IKBKB, IL1A, IL1B, JUN, MMP2, MMP9, NOS3, PLAT, MAPK8, RELA, CCL2, SELE, TNF, TP53, VCAM1
hsa05417	Lipid and atherosclerosis	28	40.00	2.372E-42	AKT1, BCL2, CASP3, CHUK, MAPK14, FOS, GSK3B, ICAM1, IKBKB, IL1B, IL6, CXCL8, JUN, MMP9, NFKBIA, NOS3, PPARG, PRKCA, MAPK1, MAPK8, RELA, CCL2, SELE, SELP, STAT3, TNF, TP53, VCAM1
hsa05200	Pathways in cancer	32	45.71	1.166E-37	AKT1, BCL2, CASP3, CHUK, ESR2, FOS, GSK3B, GSTM1, GSTP1, HMOX1, IFNG, IKBKB, IL2, IL4, IL6, CXCL8, JUN, MMP2, MMP9, NFKBIA, NOS2, PPARD, PPARG, PRKCA, MAPK1, MAPK8, PTGS2, RELA, STAT1, STAT3, TGFB1, TP53
hsa05144	Malaria	12	17.14	9.707E-22	ICAM1, IFNG, IL1B, IL6, CXCL8, IL10, CCL2, SELE, SELP, TGFB1, TNF, VCAM1
hsa04926	Relaxin signaling pathway	14	20.00	4.930E-20	AKT1, MAPK14, FOS, JUN, MMP2, MMP9, NFKBIA, NOS2, NOS3, PRKCA, MAPK1, MAPK8, RELA, TGFB1
hsa04064	NF-kappa B signaling pathway	12	17.14	1.256E-17	AKT1, BCL2, HMOX1, IFNG, IL6, NOS2, NOS3, SERPINE1, PRKCA, MAPK1, RELA, STAT3
hsa04066	HIF-1 signaling pathway	12	17.14	2.257E-17	AKT1, BCL2, HMOX1, IFNG, IL6, NOS2, NOS3, SERPINE1, PRKCA, MAPK1, RELA, STAT3, PLAT, SPP1
hsa04068	FoxO signaling pathway	12	17.14	2.197E-16	AKT1, CAT, CHUK, MAPK14, IKBKB, IL6, IL10, MAPK1, MAPK8, STAT3, TGFB1, SIRT1
hsa04917	Prolactin signaling pathway	10	14.29	7.887E-16	AKT1, MAPK14, ESR2, FOS, GSK3B, MAPK1, MAPK8, RELA, STAT1, STAT3

### Molecular docking verification

**Fig. 5:**
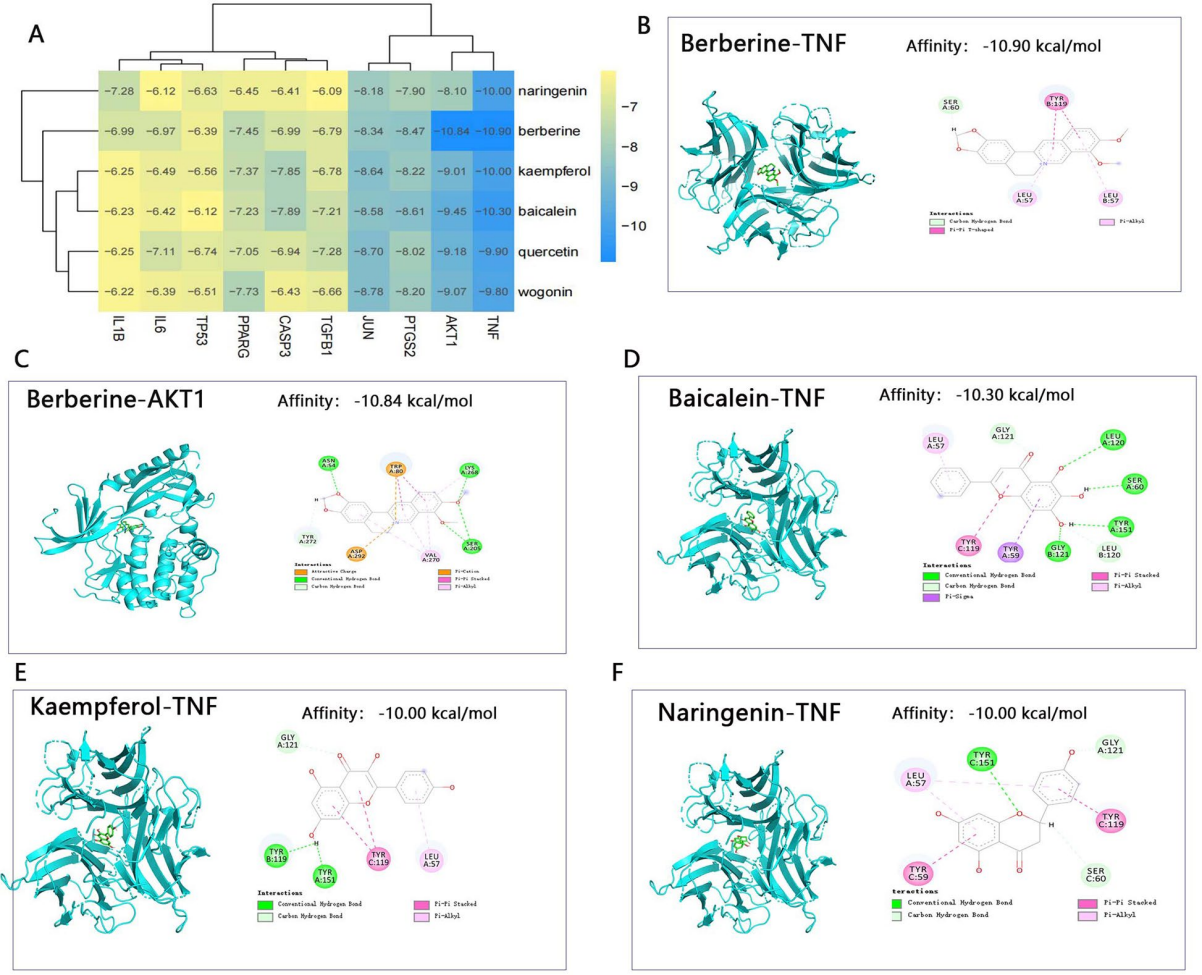
Results of molecular docking verification between the 6 core compounds of GGQLD and the 10 hub targets. **A** Heatmap of affinities. **B** Molecular docking of TNF and berberine. **C** Molecular docking of AKT1 and berberine. **D** Molecular docking of TNF and baicalein. **E** Molecular docking of TNF and kaempferol. **F** Molecular docking of TNF and naringenin

Six core compounds in GGQLD, namely, quercetin, naringenin, kaempferol, baicalein, wogonin and berberine were selected for molecular docking verification with TNF, IL6, AKT1, IL1B, PTGS2, PPARG, TP53, JUN, CASP3, and TGFB1. The affinities between the chemical compounds and the hub targets were calculated. Commonly, an affinity of < -4.25 kcal•mol-1 indicates binding activity between the chemical component and the target, an affinity of < -5.0 kcal•mol-1 implies good binding activity, and an affinity < -7.0 kcal•mol-1 suggests strong binding activity. In this study, all 6 core compounds of GGQLD had good binding activity with at least one of the 10 hub targets (Fig. 5A). TNF, AKT1 and JUN had strong affinities for all the core compounds. The 5 pairs with the best binding activity were TNF-berberine (affinity < -10.90 kcal•mol-1, Fig. 5B), AKT1-berberine (affinity < -10.84 kcal•mol-1, Fig. 5C) kcal•mol-1, TNF-baicalein (affinity < -10.30 kcal•mol-1, Fig. 5D), TNF-kaempferol (affinity < -10.00 kcal•mol-1, Fig. 5E) and TNF-naringenin (affinity < -10.00, Fig. 5F).

Analysis of RAGE docking with the 6 core compounds revealed that all the core compounds of GGQLD exhibited strong binding activity with RAGE (Table 5). The affinities were 7.83 kcal•mol-1 (RAGE-berberine), 7.76 kcal•mol-1 (RAGE-quercetin), 7.66 kcal•mol-1 (RAGE-kaempferol), 7.59 kcal•mol-1 (RAGE-baicalein), 7.47 kcal•mol-1 (RAGE-naringenin) and 7.15 kcal•mol-1 (RAGE-wogonin). The results of molecular docking verification strongly supported that the mechanism by which GGQLD alleviates the symptoms of T2DM with IBD is related to the AGE-RAGE pathway.

**Table 5:** Molecular docking verification between the 6 core compounds and RAGE

No	Docking compounds	Mol ID	molecular docking Structure	Affinity (kcal/mol)
1	berberine	MOL001454		-7.83
2	quercetin	MOL000098		-7.76
3	kaempferol	MOL000422		-7.66
4	baicalein	MOL002714		-7.59
5	naringenin	MOL004328		-7.47
6	wogonin	MOL000173		-7.15

### Identification of the chemical compounds in GGQLD via UHPLC-MS

**Fig. 6:**
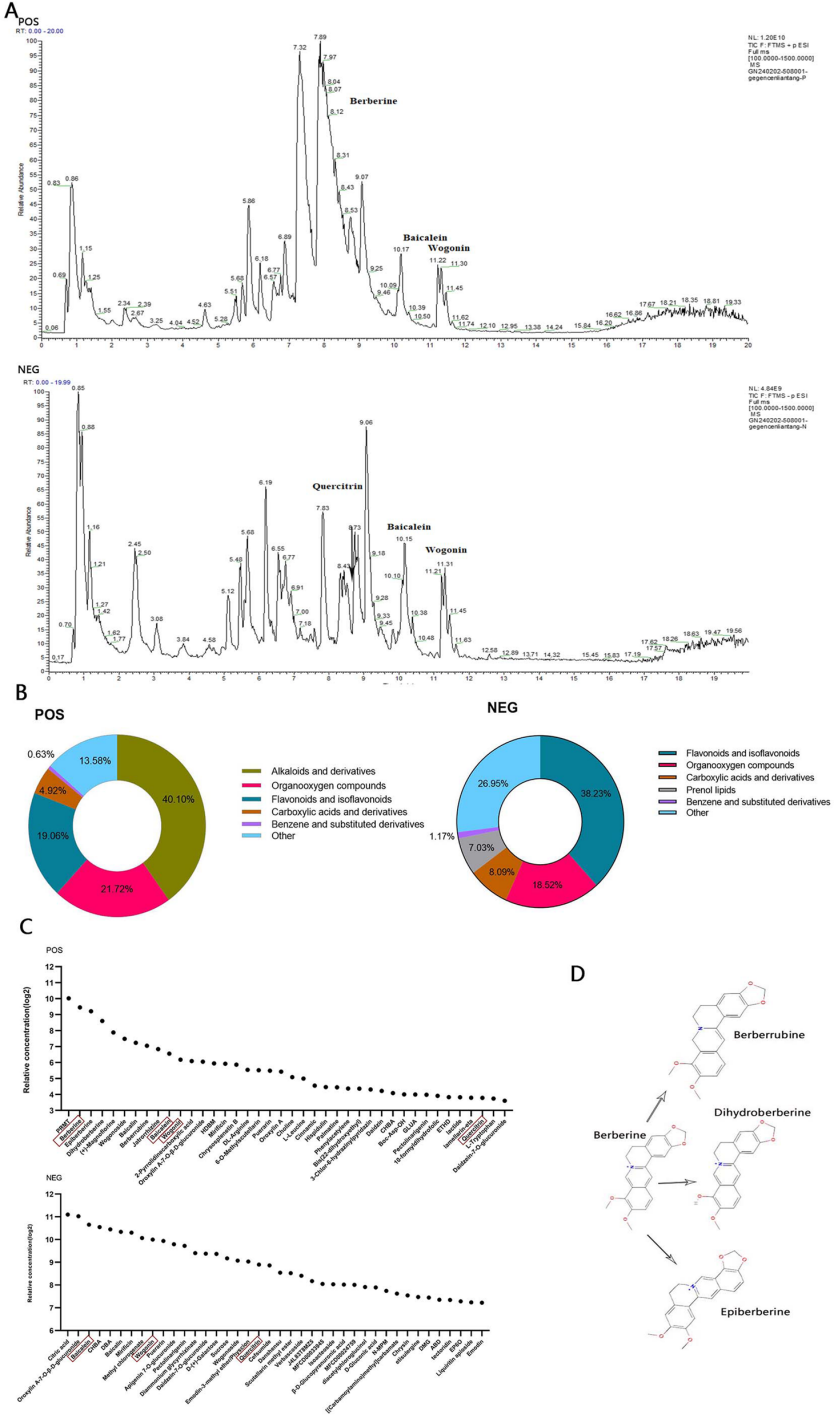
The chemical compounds in GGQLD. **A** Total ion chromatogram for GGQLD obtained via UHPLC-MS. **B** The relative percentages of different compound classes. **C** The chemical compounds with the 20 highest relative concentrations in GGQLD. **D** Berberine and 3 derivatives; POS: positive ion mode, NEG: negative ion mode

To verify the above results by a network pharmacology approach, we used UHPLC-MS to determine the relative concentrations of chemical compounds in GGQLD. In total, 238 chemical compounds were detected in positive ion mode, 162 chemical compounds were detected in negative ion mode, and 52 compounds were detected in both modes (File S4, Fig. 6A). Finally, 348 chemical compounds were identified in this study.

Alkaloids and derivatives (40.10%), organooxygen compounds (27.12%) and flavonoids and isoflavonoids (19.06%) were the 3 most abundant classes of compounds detected in positive ion mode, and flavonoids and isoflavonoids (38.23%), organooxygen compounds (18.52%) and carboxylic acids and derivatives (8.09%) were the 3 most abundant classes of compounds detected in negative ion mode (Fig. 6B). All 6 core compounds identified by network pharmacology analysis with TCMSP were detected in this verification experiment. Berberine and its derivatives were the most important active compounds of GGQLD, and the percentage of berberine was highest (14.19%) in the positive ion mode (Table 6, Fig. 6C-D). Flavonoids, including baicalein (5.67%), baicalin (4.56%), wogonin (3.60%), wogonoside (1.89%) and quercitrin (1.68%), were the most vital compounds of GGQLD in negative ion mode (Table 6, Fig. 6C). Kaempferol and naringenin, which were identified as core compounds by network pharmacology, were also detected in positive ion mode, but the percentages were only 0.02% and 0.01%, respectively (File S4). The results of this experiment partially supported the results of network pharmacology analysis and revealed that the core compounds, including berberine, baicalein, wogonin, and quercitrin, are the main active compounds of GGQLD, but the concentrations of kaempferol and naringenin in GGQLD are low.

**Table 6:** Characterization of the main chemical compounds in GGQLD with UHPLC-MS

No. POS	Name	Annotation MW	Calc. MW	RT [min]	Area	Relative Percentage	Class
1	(4-Methoxyphenyl)[6-(4-methoxyphenyl)-3-pyridinyl]methanone	319.12	319.12	7.89	1.3320E + 11	21.05%	Organooxygen compounds
2	Berberine	335.12	335.11	8.14	8.9806E + 10	14.19%	Alkaloids and derivatives
3	Epiberberine	335.12	335.11	8.43	7.6186E + 10	12.04%	Alkaloids and derivatives
4	Dihydroberberine	337.13	337.13	7.39	4.9999E + 10	7.90%	Alkaloids and derivatives
5	( +)-Magnoflorine	341.16	341.16	5.86	3.0337E + 10	4.79%	Aporphines
6	Wogonoside	460.10	460.10	9.07	2.3036E + 10	3.64%	Flavonoids
7	Baicalin	446.08	446.08	7.81	1.9352E + 10	3.06%	Flavonoids
8	Berberrubine	321.10	321.10	6.88	1.7054E + 10	2.70%	Alkaloids and derivatives
9	Jatrorrhizine	337.13	337.13	7.23	1.4749E + 10	2.33%	Alkaloids and derivatives
10	Baicalein	270.05	270.05	10.16	1.2093E + 10	1.91%	Flavonoids
11	Wogonin	284.07	284.07	11.22	9.3095E + 09	1.47%	Flavonoids
12	2-Pyrrolidinecarboxylic acid	115.06	115.06	0.88	8.7476E + 09	1.38%	Carboxylic acids and derivatives
13	Oroxylin A-7-O-β-D-glucuronide	460.10	460.10	8.74	8.5488E + 09	1.35%	Flavonoids
14	N-(4-Hydroxy-3-nitrophenyl)-2-[(1-methyl-2,4,6-trioxohexahydro-5-pyrimidinyl)carbonyl]hydrazinecarboxamide	380.07	380.07	0.82	7.9169E + 09	1.25%	Others
15	Mirificin	548.15	548.15	6.77	7.8033E + 09	1.23%	Prenol lipids
16	Chrysosplenetin B	374.10	374.10	11.31	7.4935E + 09	1.18%	Flavonoids
17	DL-Arginine	174.11	174.11	0.83	6.0034E + 09	0.95%	Carboxylic acids and derivatives
18	6-O-Methylscutellarin	476.10	476.09	8.84	5.9041E + 09	0.93%	Flavonoids
19	Puerarin	416.11	416.11	5.68	5.7739E + 09	0.91%	Isoflavonoids
20	Oroxylin A	284.07	284.07	11.45	5.5433E + 09	0.88%	Flavonoids
NEG							
1	Citric acid	192.03	192.03	1.16	1.1016E + 10	7.71%	Carboxylic acids and derivatives
2	Oroxylin A-7-O-β-D-glucuronide	460.10	460.10	9.07	1.0468E + 10	7.32%	Flavonoids
3	Baicalein	270.05	270.05	10.17	8.1044E + 09	5.67%	Flavonoids
4	(3R,5R)-1,3,5-Trihydroxy-4-{[(2E)-3-(4-hydroxy-3-methoxyphenyl)-2-propenoyl]oxy}cyclohexanecarboxylic acid	368.11	368.11	6.19	7.5289E + 09	5.27%	Organooxygen compounds
5	4-(beta-D-Glucopyranosyloxy)-3,5-dimethoxybenzoic acid	360.11	360.11	2.46	7.0192E + 09	4.91%	Tannins
6	Baicalin	446.08	446.08	7.82	6.5168E + 09	4.56%	Flavonoids
7	Mirificin	548.15	548.15	6.77	6.3536E + 09	4.45%	Prenol lipids
8	Methyl chlorogenate	368.11	368.11	5.46	5.3908E + 09	3.77%	Organooxygen compounds
9	Wogonin	284.07	284.07	11.22	5.1430E + 09	3.60%	Flavonoids
10	Puerarin	416.11	416.11	5.67	4.9304E + 09	3.45%	Isoflavonoids
11	Apigenin 7-O-glucuronide	446.08	446.08	8.43	4.4589E + 09	3.12%	Flavonoids
12	Pectolinarigenin	314.08	314.08	11.32	4.2502E + 09	2.97%	Flavonoids
13	Diammonium glycyrrhizinate	822.40	822.40	10.10	3.4081E + 09	2.38%	Prenol lipids
14	Daidzein-7-O-glucuronide	430.09	430.09	8.84	3.3416E + 09	2.34%	Isoflavonoids
15	D-( +)-Galactose	180.06	180.06	0.83	3.3247E + 09	2.33%	Organooxygen compounds
16	Sucrose	342.12	342.12	0.82	2.8986E + 09	2.03%	Organooxygen compounds
17	Wogonoside	460.10	460.10	8.74	2.7045E + 09	1.89%	Flavonoids
18	Emodin-3-methyl ether/Physcion	284.07	284.07	11.45	2.6352E + 09	1.84%	Anthracenes
19	Quercitrin	448.10	448.10	8.33	2.4028E + 09	1.68%	Flavonoids
20	Cefsumide	440.08	440.08	0.94	2.3417E + 09	1.64%	Lactams

^*^ *MW* Molecular weight, *Calc. MW* Molecular weight was obtained by this study, *RT* Retention time, *POS* Positive ion mode, *NEG* Negative ion mode

### GGQLD treatment decreased blood glucose in rats with T2DM and IBD

**Fig. 7:**
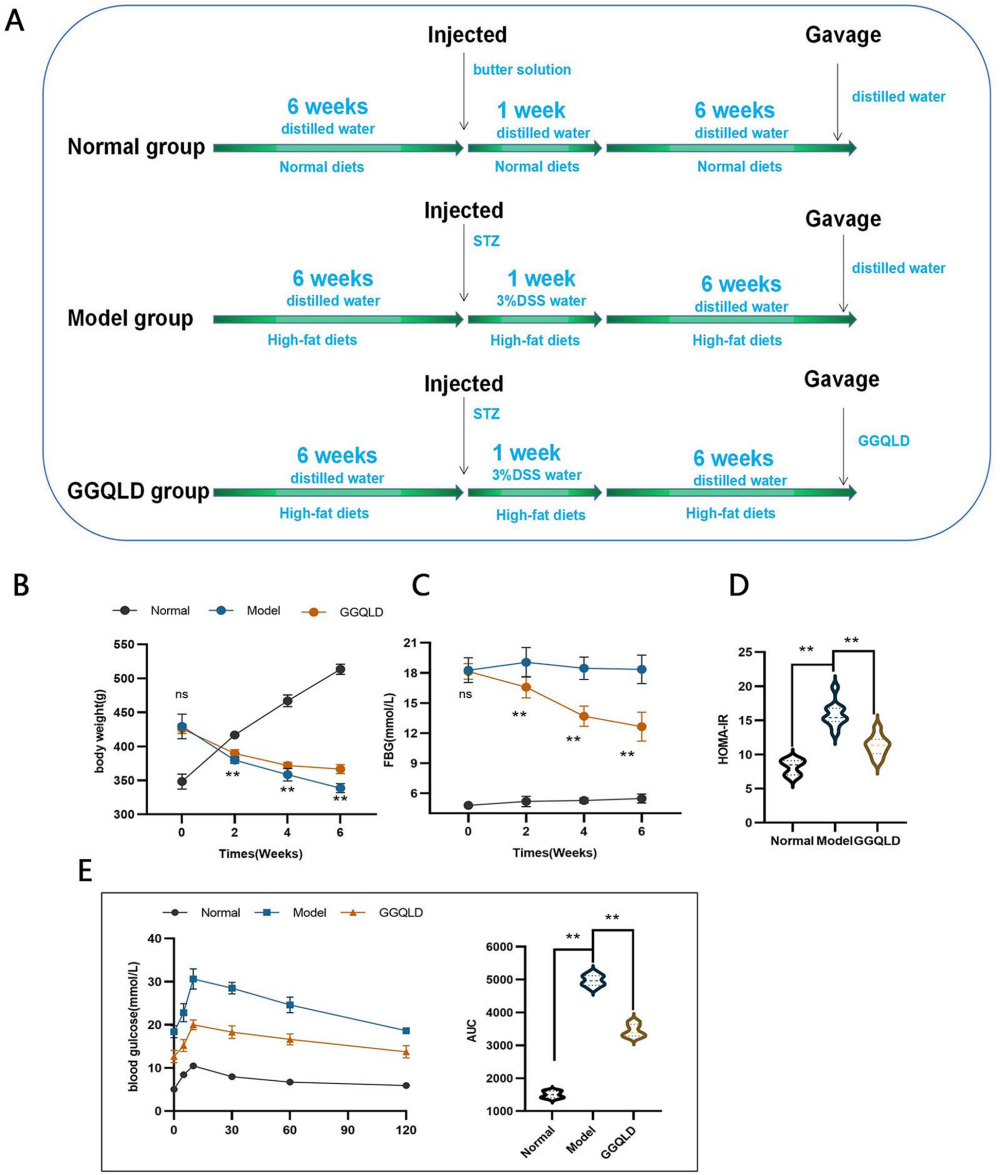
Therapeutic effects of GGQLD on T2DM rats. **A** Design of the animal experiment. **B** Body weight. **C** Fasting blood glucose (FBG). **D** Homeostatic model assessment for insulin resistance (HOMA-IR) index. **E** Blood glucose measurement in the oral glucose tolerance test (OGTT) and area under the curve (AUC) for the OGTT. *n* = 10 rats per group. The data are presented as the means ± SDs. Statistical analysis was performed using Student’s t test. Compared to the model group, *p < 0.05, **p < 0.01, ns: *p* > 0.05

A rat model of T2DM and IBD was established by the administration of STZ and DSS (Fig. 7A). The body weight decreased continuously after the model was established, and GGQLD treatment reduced the decrease in body weight (Fig. 7B). Many studies have shown that GGQLD can alleviate the symptoms of T2DM [25, 35]. Our study supported the findings of previous studies and revealed that GGQLD obviously decreased FBG by 12.96% (after 2 weeks), 25.89% (after 4 weeks) and 31.12% (after 6 weeks) (Fig. 7C). The HOMA-IR index, which reflects insulin resistance, decreased by 14.94% after 6 weeks of GGQLD treatment (Fig. 7D). The OGTT is usually used to assess glucose intolerance; GGQLD prevented a substantial increase in blood glucose during the OGTT, and the AUC decreased markedly, by 34.85% (Fig. 7E).

### GGQLD ameliorated intestinal inflammation in rats with T2DM and IBD

**Fig. 8:**
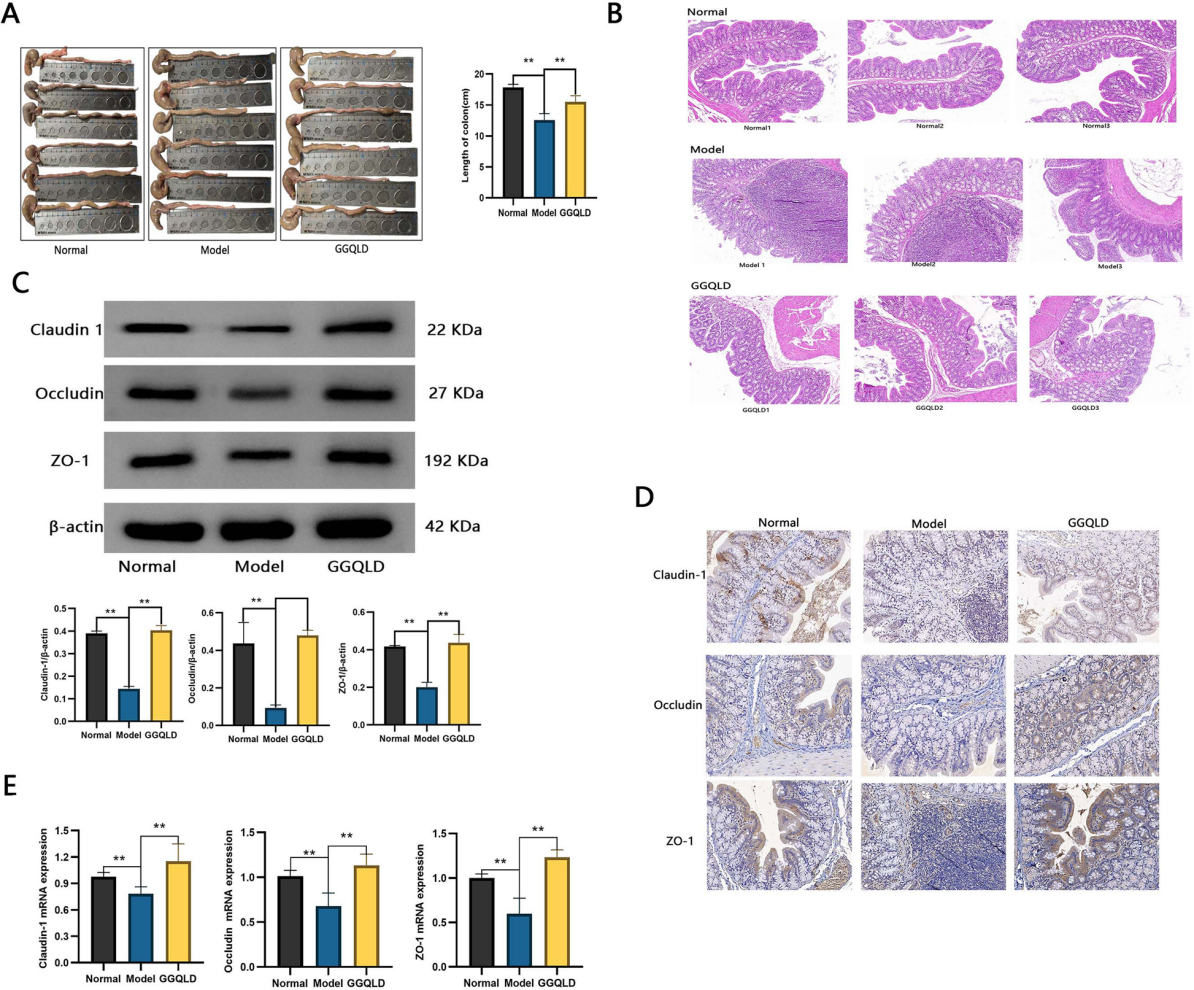
Therapeutic effects of GGQLD on intestinal inflammation in T2DM rats with IBD. **A** Length of the colon. **B** H&E staining of the colon. **C** Western blot analysis of claudin-1, occludin and ZO-1. **D** Immunohistochemical staining for claudin-1, occludin and ZO-1. **E** Relative mRNA expression of claudin-1, occludin and ZO-1. The data are presented as the means ± SDs. Statistical analysis was performed using Student’s t test. Compared to the model group, **p < 0.01

GGQLD attenuated colonic erythema, swelling, bleeding, and shortening in the model rats (Fig. 8A). H&E staining revealed that GGQLD effectively reversed the damage to the villous and crypt morphology in the colon (Fig. 8B). Claudin-1, occludin and ZO-1 are the most important tight junction proteins in the intestinal mucosa, and their expression levels represent the integrity of the intestinal barrier. The protein and mRNA expression levels of claudin-1, occludin and ZO-1 were significantly increased by GGQLD treatment (Fig. 8C-E, FigS1A).

### The AGE-RAGE pathway was regulated by GGQLD

**Fig. 9:**
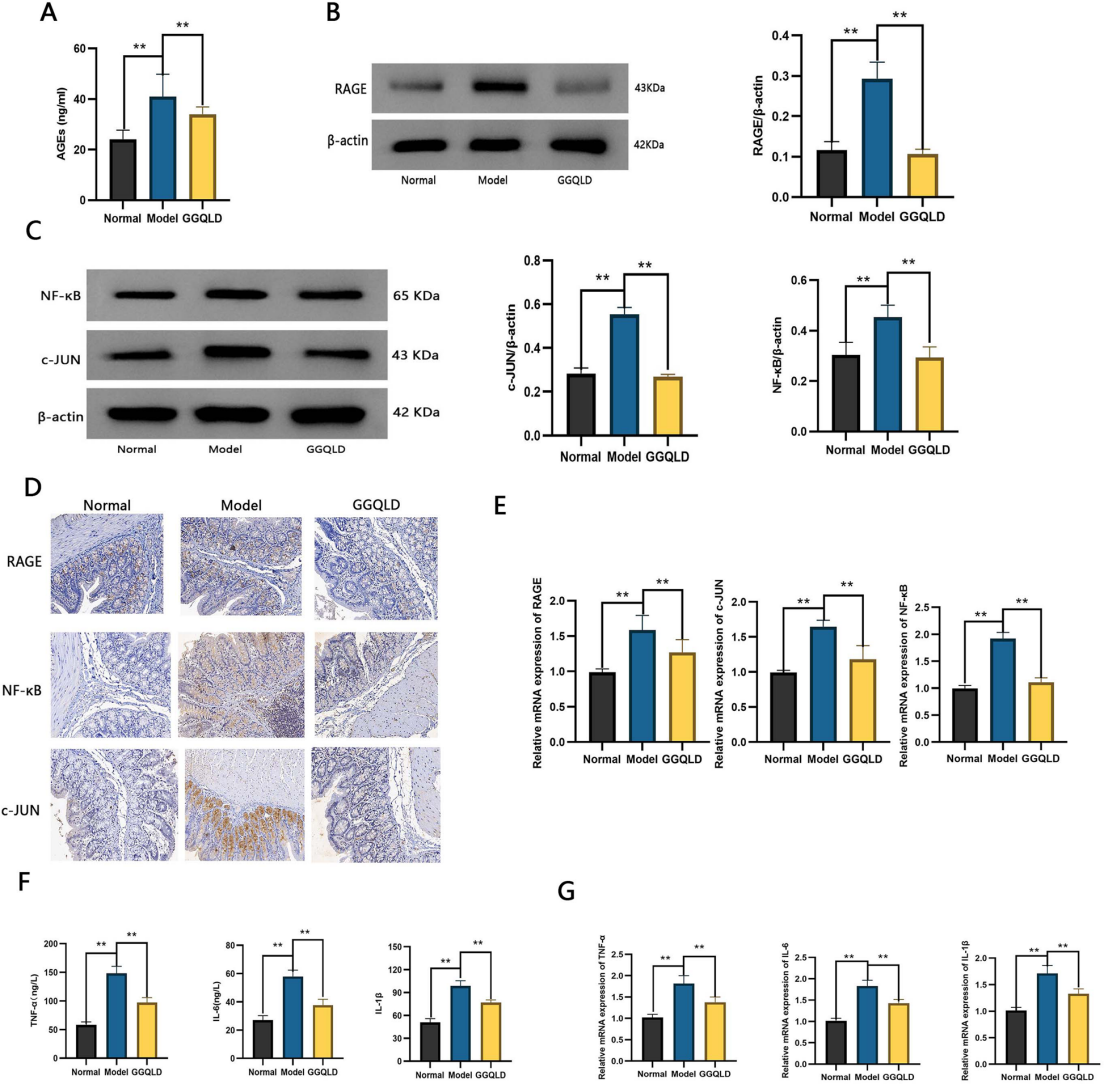
Effects of GGQLD on the AGE-RAGE pathway. **A** The concentration of AGEs. **B** Western blot analysis of RAGE. **C** Western blot analysis of c-JUN and NF-κB. **D** Immunohistochemical staining for RAGE, c-JUN and NF-κB. **E** Relative mRNA expression of RAGE, c-JUN and NF-κB. **F** Concentrations of TNF-α, IL-6 and IL-1β. **G** Relative mRNA expression of TNF-α, IL-6 and IL-1β.The data are presented as the means ± SDs. Statistical analysis was performed using Student’s t test. Compared to the model group, **p < 0.01

The network pharmacology and molecular docking results revealed that the mechanism of GGQLD in treating T2DM with IBD is related to the AGE-RAGE pathway. In this part of the study, the above hypothesis was proven through animal experiments. The concentration of AGEs decreased greatly in response to GGQLD (Fig. 9A). Correspondingly, the expression of RAGE, NF-κB and c-JUN was reduced by GGQLD treatment (Fig. 9B-E, FigS1B-C). The levels of proinflammatory cytokines, including TNF-α, IL-6 and IL-1β, were significantly decreased after GGQLD treatment (Fig. 9F-G). The above results verified the network pharmacology and molecular docking results and revealed that the anti-inflammatory effects of GGQLD were mediated via the AGE-RAGE pathway.

## Discussion

T2DM is characterized by hyperglycemia and results from insulin resistance and insufficient insulin secretion, and it is one of the most common chronic diseases in humans. Although many drugs exist to control blood glucose levels, their therapeutic effects on T2DM are unsatisfactory [36]. Hence, further research on novel therapies for T2DM is urgently needed. Recently, accumulating evidence has indicated the crucial pathogenic role of the chronic low-grade inflammatory response in insulin resistance and the close relationship between T2DM and intestinal inflammation [11]. High-fat and high-sugar diets result in destruction of the intestinal barrier, which leads to the entry of materials from the intestine into the bloodstream and the production of proinflammatory cytokines [5]. These proinflammatory cytokines immediately lead to the development of IBD and prevent insulin signal transduction [5]. Therefore, inhibiting intestinal inflammation is important not only for the treatment of IBD but also for the improvement of insulin resistance.

In this study, GGQLD, a classical Chinese medicinal formula that has often been reported for its ability to reduce blood glucose and alleviate intestinal inflammation, was used to treat T2DM with IBD in rats [35]. Network pharmacology analysis and molecular docking validation were used to explore the potential mechanisms of GGQLD in treating T2DM with IBD. Six core compounds in GGQLD, namely, quercetin, naringenin, kaempferol, baicalein, wogonin and berberine, and 70 potential therapeutic targets through which GGQLD alleviates T2DM with IBD were selected. Finally, the PPI network analysis results showed that all the hub targets, namely, TNF, IL6, AKT1, IL1B, PTGS2, PPARG, TP53, JUN, CASP3, and TGFB1, were closely related to the inflammatory response. The above results strongly supported our hypothesis that GGQLD alleviates symptoms of T2DM by regulating intestinal inflammation.

Furthermore, KEGG pathway enrichment analysis revealed that the AGE-RAGE signaling pathway in diabetic complications was the most important pathway involved in the therapeutic effect of GGQLD on T2DM with IBD. Hyperglycemia and hyperlipidemia induce the accumulation of AGEs, and clinical studies have also shown that the concentration of AGEs is greater in diabetic patients [37]. RAGE is now regarded as a representative AGE receptor on the endothelium. RAGE regulates the expression of the cytokines and oxidative stress via activation of c-JUN/NF-κB [38, 39].c-JUN and NF-κB are transcription factors that regulate the transcription of different genes involved in the expression of inflammatory cytokines (including TNF-α, IL-1β, IL-6 and IL-10) [40, 41]. As we described above, proinflammatory cytokines result in insulin resistance [5]. AGE-RAGE-mediated chronic inflammation controls the pathophysiological process of diabetic complications [39, 42]. Intestinal inflammation in patients with T2DM is one such diabetic complication.Recently, an increasing number of studies have shown that inhibiting the expression of RAGE can alleviate intestinal inflammation [43]. Compared with WT mice, *Rage-/-* mice are less susceptible to intestinal and colonic inflammation, and the expression of c-JUN and NF-κB in the intestine decreases after the expression of RAGE is blocked [43–45]. Although we found that almost half of the T2DM patient population experiences intestinal symptoms, studies on T2DM complicated with IBD are lacking. Whether the AGE-RAGE pathway influences the process of intestinal inflammation in T2DM patients and the related mechanisms underlying the therapeutic effect of GGQLD on T2DM with IBD need further exploration.

Molecular docking studies confirmed the above results; the 6 GGQLD core compounds had good affinity not only for the 10 hub targets but also for RAGE. The results of network pharmacology analysis and molecular docking validation revealed that the mechanism by which GGQLD alleviates T2DM with IBD might be related to the AGE-RAGE pathway. However, the limitations of the above research are obvious. All the core compounds, targets and signaling pathways were obtained from online databases, and the core target, RAGE, was not included in any of these databases; thus, we could not obtain data for RAGE. Furthermore, all of the above results were confirmed by bioinformatics methods, and the real-world results are unknown.

To overcome the above limitations, UHPLC-MS was used to identify the chemical compounds in GGQLD, and an animal experiment was used to evaluate the therapeutic effects of GGQLD on T2DM with IBD and the regulatory effects of GGQLD on the AGE-RAGE pathway. All 6 core compounds were detected by UHPLC-MS and among the 6 core compounds, berberine had the highest relative concentration in GGQLD. Baicalein, wogonin and quercitrin were also determined to be important compounds in GGQLD, but the concentrations of kaempferol and naringenin in GGQLD were low. This finding indicated that the most important active compound in GGQLD must be berberine and that baicalein, wogonin and quercitrin are also indispensable.

Some studies have confirmed our results: berberine is the main component of Huanglian that decreases blood glucose and lipids and alleviates intestinal inflammation [23, 46]. Repairing the intestinal barrier and regulating the composition of the gut microbiota are considered the crucial mechanisms underlying the therapeutic effect of berberine on T2DM and IBD [47, 48]. Baicalein, the most important active compound in Huangqin, has been reported to have antineoplastic and anti-inflammatory effects, improving insulin resistance [49, 50]. Studies have shown that baicalein can suppress high glucose-induced inflammation and ameliorate ulcerative colitis by strengthening the intestinal epithelial barrier [50, 51]. Wogonin is the main component of Huangqin, and quercitrin is the main component of both Huanglian and Gancao. All of these compounds can ameliorate inflammation in T2DM patients [52, 53]. In our UHPLC-MS experiment, a total of 348 chemical compounds were identified in GGQLD. Many of these compounds contribute to the therapeutic effects of GGQLD on T2DM, but the most important mechanism underlying the therapeutic effect of GGQLD on T2DM involves controlling inflammation via the core compounds berberine, baicalein, wogonin and quercitrin.

The therapeutic effects of GGQLD on T2DM with IBD were verified by experiments in a rat model. GGQLD obviously reduced blood glucose levels and ameliorated intestinal inflammation (Figs. 7 and 8). The concentration of AGEs decreased in response to GGQLD (Fig. 9). Correspondingly, the expression of RAGE was downregulated by GGQLD (Fig. 9). As mentioned before, the activation of RAGE can activate the expression of c-JUN/NF-κB, leading to the production of more proinflammatory cytokines (Fig. 9). In our study, after the AGE-RAGE pathway was blocked by GGQLD, the expression of c-JUN/NF-κB decreased (Fig. 9). Therefore, the expression of inflammatory intestinal cytokines, such as TNF-α, IL-6 and IL-1β, was greatly decreased by GGQLD (Fig. 9). The intestinal barrier was subsequently restored by the inhibition of intestinal inflammation and the upregulation of junction proteins (Fig. 8). Some studies supported our results: Huanglian, a core herb of GGQLD, was found to alleviate eczema by suppressing AGE-RAGE, downregulating the expression of c-JUN/NF-κB and inhibiting the proinflammatory signaling pathway [54];berberine, the most important chemical component of GGQLD, exerted renoprotective effects by regulating the AGE-RAGE signaling pathway in diabetic nephropathy [55].

## Conclusion

**Fig. 10:**
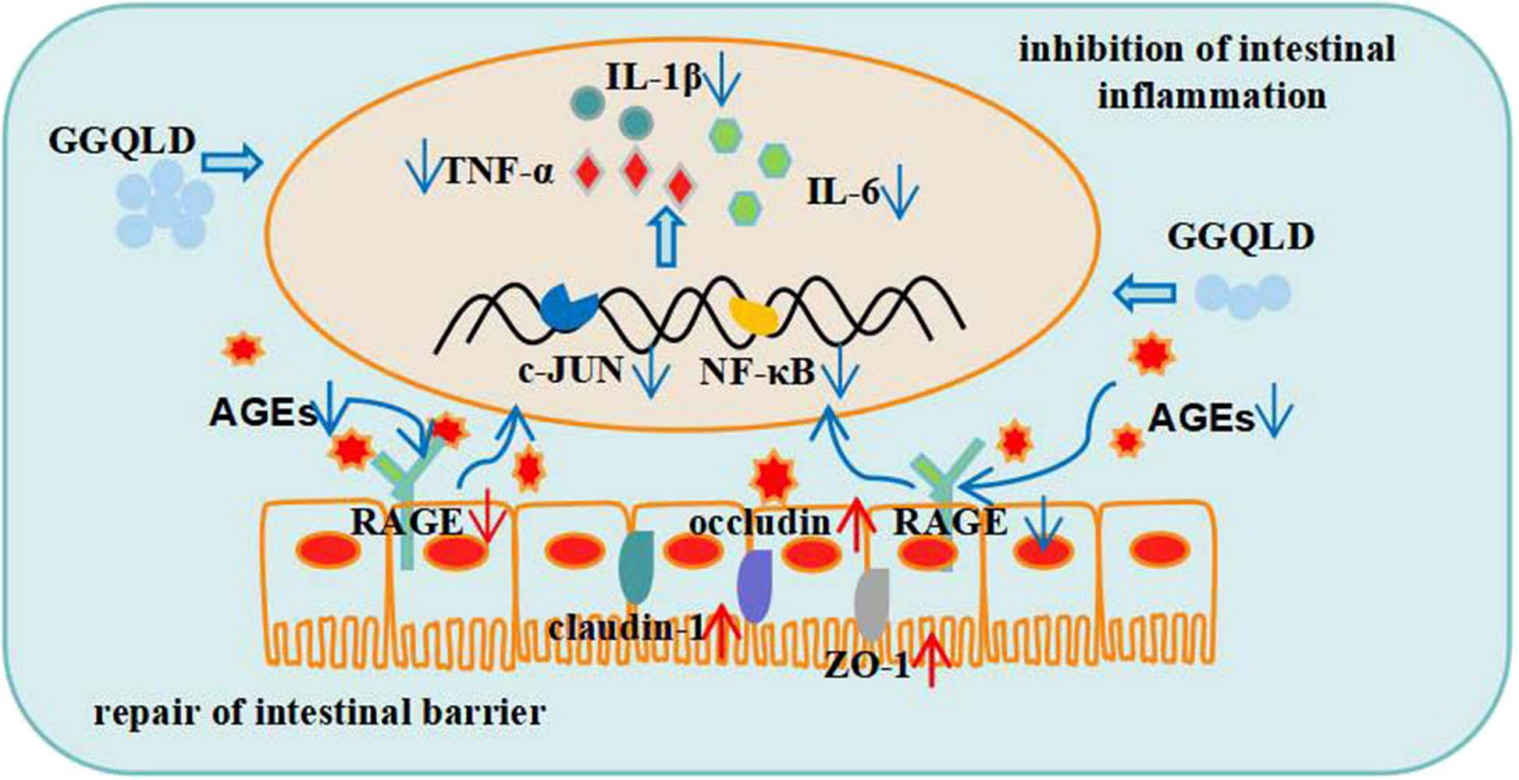
The mechanism of GGQLD alleviates the symptoms of T2DM with IBD

Most T2DM patients exhibit intestinal symptoms, and GGQLD is an effective Chinese medicine for treating both T2DM and IBD. Then, network pharmacology analysis and molecular docking validation were used to explore the potential mechanisms of GGQLD in treating T2DM with IBD. UHPLC-MS and animal experiments were then performed to verify the above results. Finally, we found that berberine, baicalein, wogonin and quercitrin were the most important active compounds of GGQLD for the treatment of T2DM and that AGE-RAGE pathway-related anti-inflammatory mechanisms play crucial roles in the ability of GGQLD to ameliorate the symptoms of T2DM with IBD (Fig. 10).

### Supplementary Information

Additional file 1: Fig S1: Supplementary material for western blotting

Additional file 2: Table S1: The 146 ingredients of GGQLD

Additional file 3: File S1: Supplementary methods

Additional file 4: File S2: Herb-compoents-targets

Additional file 5: File S3: T2DM-IBD-targets

Additional file 6: File S4: Chemical compounds of GGQLD

## Data Availability

The original contributions presented in the study are included in the article/Supplementary Material. Further inquiries can be directed to the corresponding authors.

## Abbreviations

AGEs: Advanced glycation end products
AUC: Area under the curve
BC: Betweenness centrality
BG: Blood glucose
BP: Biological process
CC: Cellular component
DEGs: Differentially expressed genes
DL: Drug-likeness
FBG: Fasting blood glucose
GC: Gancao
GG: Gegen
GGQLD: Ge-Gen-Qin-Lian decoction
H&E staining: Hematoxylin and eosin staining
HL: Huanglian
HOMA-IR: Homeostasis model assessment of insulin resistance index
HQ: Huangqin
IL-1β: Interleukin1β
IL-6: Interleukin 6
MF: Molecular function
NCBI: National Center for Biotechnology Information
NEG: Negative ion mode
OB: Oral bioavailability
OGTT: Oral glucose tolerance test
POS: Positive ion mode
PPI: Protein–protein interaction
PVDF: Polyvinylidene fluoride
qRT-PCR: Quantitative real-time PCR
RAGE: Receptor for advanced glycation end products
SD rat: Sprague–Dawley rat
T2DM: Type 2 diabetes mellitus
TNF-α: Tumor necrosis factor-α
UHPLC-MS: Ultra-high performance liquid chromatography-mass spectrometry
