# Supplementary material for: Ge-Gen-Qin-Lian decoction alleviates the symptoms of type 2 diabetes mellitus with inflammatory bowel disease via regulating the AGE-RAGE pathway
Source: BMC Complement Med Ther. 2024 Jun 10;24:225. doi: 10.1186/s12906-024-04526-x (PMC11163797; doi:10.1186/s12906-024-04526-x)
Supplement: Supplementary file 1 — Additional file 1: Fig S1: Supplementary material for western blotting [file 12906_2024_4526_MOESM1_ESM.pdf]

Fig S1. Supplementary material for Western Blotting

A

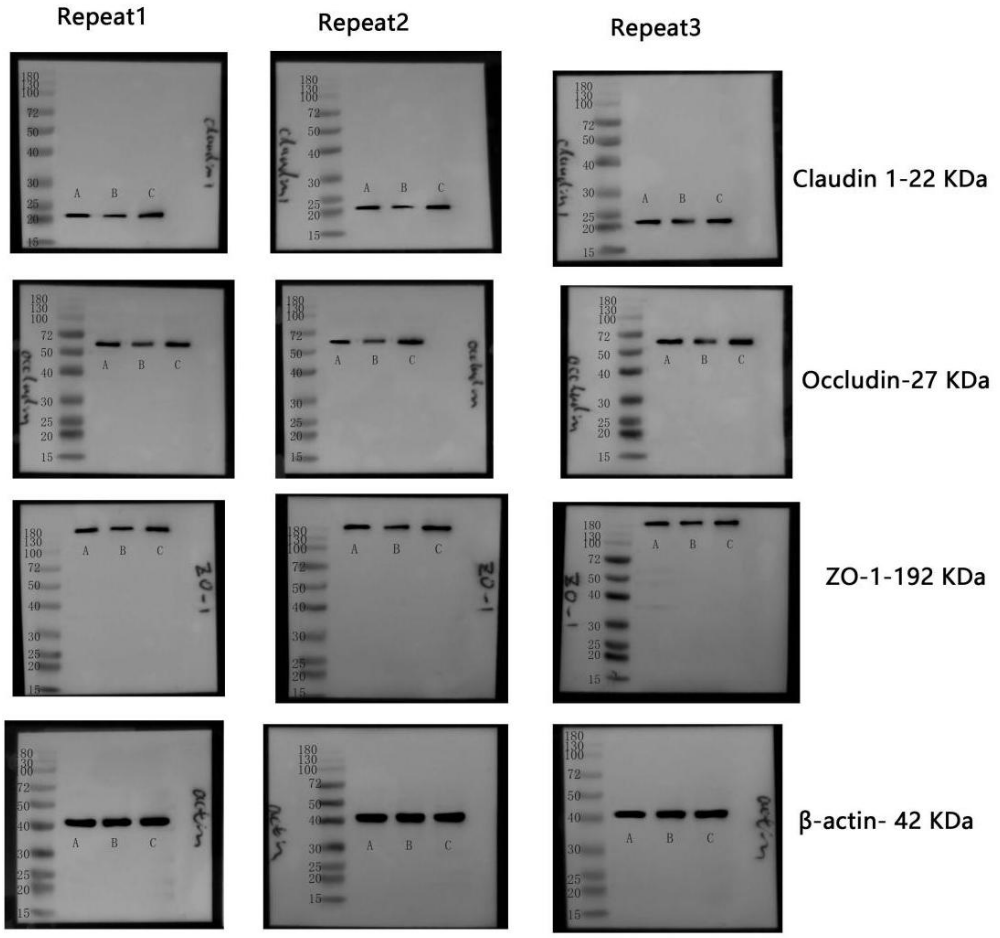

B

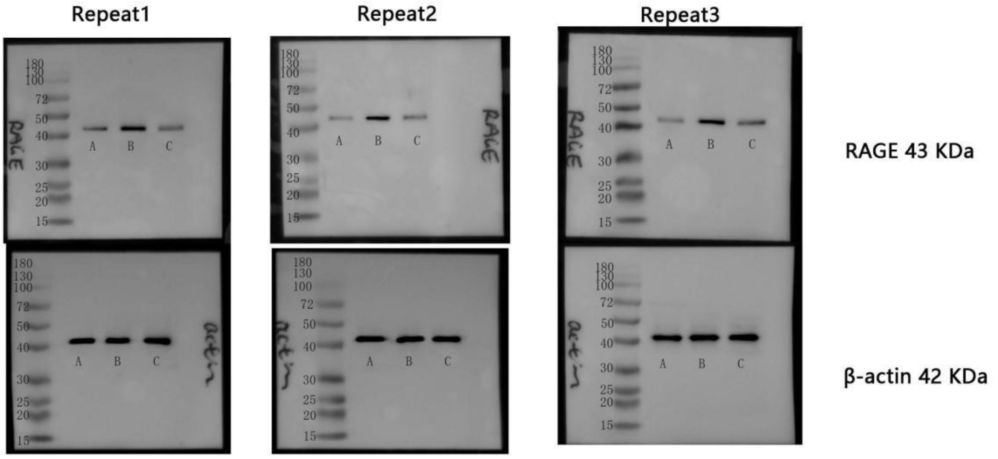

C

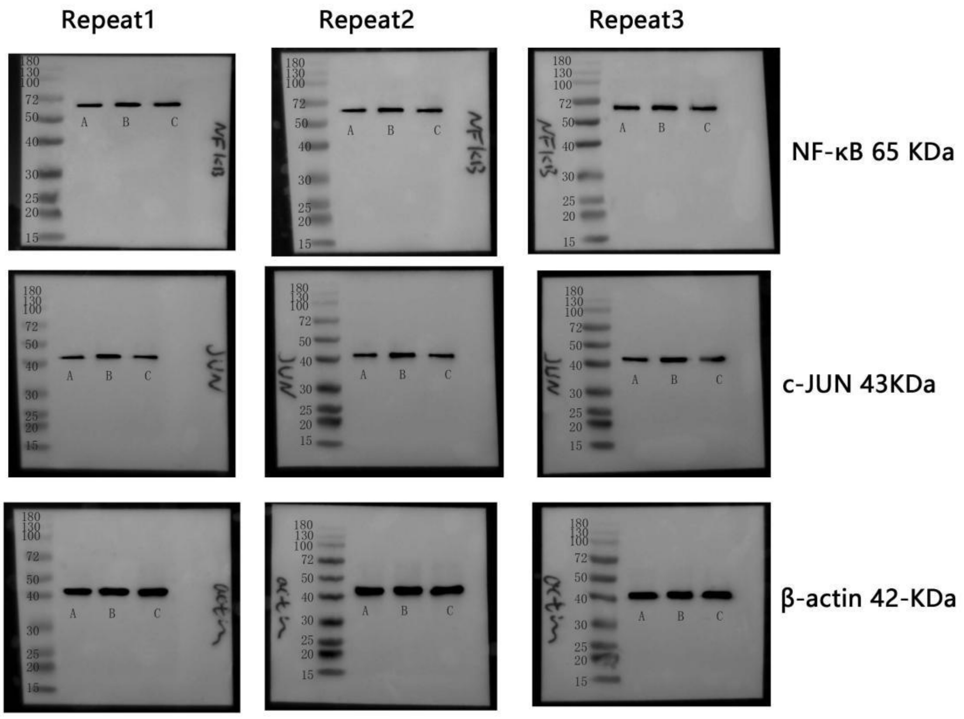

Fig S1. Supplementary material for Western Blotting. A: Original Western blotting of claudin-1, occludin and ZO-1; B: Original Western blotting of RAGE; C: Original Western blotting of NF-κB and c-JUN. A: normal group; B: model group; C:GGQLD group.
