# Supplementary material for: Ge-Gen-Qin-Lian decoction alleviates the symptoms of type 2 diabetes mellitus with inflammatory bowel disease via regulating the AGE-RAGE pathway
Source: BMC Complement Med Ther. 2024 Jun 10;24:225. doi: 10.1186/s12906-024-04526-x (PMC11163797; doi:10.1186/s12906-024-04526-x)
Supplement: Supplementary file 2 — Additional file 2: Table S1: The 146 ingredients of GGQLD [file 12906_2024_4526_MOESM2_ESM.pdf]

TableS1. The 146 compounds of GGQLD.

ID	MOL ID	Molecule Name	Molecular Weight	Source	ID	MOL ID	Molecule Name	Molecular Weight	Source
A	MOL000392	formononetin	268.28	Gegen	GC20	MOL004814	Isotrifoliol	298.26	Gancao
B	MOL000358	beta-sitosterol	414.79	Gegen	GC21	MOL004815	(E)-1-(2,4-dihydroxyphenyl)-3-(2,2-dimethylchromen-6-yl)prop-2-en-1-one	322.38	Gancao
GG3	MOL002959	3'-Methoxydaidzein	284.28	Gegen	GC22	MOL004820	kanzonols W	336.36	Gancao
GG4	MOL003629	Daidzein-4,7-diglucoside	578.57	Gegen	GC23	MOL004824	(2S)-6-(2,4-dihydroxyphenyl)-2-(2-hydroxypropan-2-yl)-4-methoxy-2,3-dihydrofuro[3,2-g]chromen-7-one	384.41	Gancao
HL1	MOL001454	berberine	336.39	Huanglian	GC24	MOL004827	Semilicoisoflavone B	352.36	Gancao
HL2	MOL013352	Obacunone	454.56	Huanglian	GC25	MOL004828	Glepidotin A	338.38	Gancao
HL3	MOL002894	berberrubine	322.36	Huanglian	GC26	MOL004829	Glepidotin B	340.4	Gancao
C	MOL002897	epiberberine	336.39	Huanglian	GC27	MOL004833	Phaseolinisoflavan	324.4	Gancao
HL5	MOL002903	(R)-Canadine	339.42	Huanglian	GC28	MOL004835	Glypallichalcone	284.33	Gancao
HL6	MOL002904	Berlambine	351.38	Huanglian	GC29	MOL004838	8-(6-hydroxy-2-benzofuranyl)-2,2-dimethyl-5-chromenol	308.35	Gancao
HL7	MOL002907	Corchoroside A_qt	404.55	Huanglian	GC30	MOL004841	Licochalcone B	286.30	Gancao
HL8	MOL000622	Magnograndiolide	266.37	Huanglian	GC31	MOL004848	licochalcone G	354.43	Gancao
HL9	MOL000762	Palmidin A	510.52	Huanglian	GC32	MOL004849	3-(2,4-dihydroxyphenyl)-8-(1,1-dimethylprop-2-enyl)-7-hydroxy-5-methoxy-coumarin	368.41	Gancao
HL10	MOL000785	palmatine	352.44	Huanglian	GC33	MOL004855	Licoricone	382.44	Gancao
D	MOL000098	quercetin	302.25	Huanglian	GC34	MOL004856	Gancaonin A	352.41	Gancao
F	MOL001458	coptisine	320.34	Huanglian	GC35	MOL004857	Gancaonin B	368.41	Gancao
HL13	MOL002668	Worenine	334.37	Huanglian	GC36	MOL004860	licorice glycoside E	693.71	Gancao
HL14	MOL008647	Moupinamide	313.38	Huanglian	GC37	MOL004863	3-(3,4-dihydroxyphenyl)-5,7-dihydroxy-8-(3-methylbut-2-enyl)chromone	354.38	Gancao
HQ1	MOL001689	acacetin	284.28	HuangQin	GC38	MOL004864	5,7-dihydroxy-3-(4-methoxyphenyl)-8-(3-methylbut-2-enyl)chromone	352.41	Gancao
HQ2	MOL000173	wogonin	284.28	HuangQin	GC39	MOL004866	2-(3,4-dihydroxyphen	354.38	Gancao

							yl)-5,7-dihydroxy-6-(3-methylbut-2-enyl)chromone		
HQ3	MOL000228	(2R)-7-hydroxy-5-methoxy-2-phenylchroman-4-one	270.30	HuangQin	GC40	MOL004879	Glycyrin	382.44	Gancao
HQ4	MOL002714	baicalein	270.25	HuangQin	GC41	MOL004882	Licocoumarone	340.40	Gancao
HQ5	MOL002908	5,8,2'-Trihydroxy-7-methoxyflavone	300.28	HuangQin	GC42	MOL004883	Licoisoflavone	354.38	Gancao
HQ6	MOL002909	5,7,2,5-tetrahydroxy-8,6-dimethoxyflavone	376.34	HuangQin	GC43	MOL004884	Licoisoflavone B	352.36	Gancao
HQ7	MOL002910	Carthamidin	288.27	HuangQin	GC44	MOL004885	licoisoflavanone	354.38	Gancao
HQ8	MOL002911	2,6,2',4'-tetrahydroxy-6'-methoxychaleone	302.30	HuangQin	GC45	MOL004891	shinpterocarpin	322.38	Gancao
HQ9	MOL002913	Dihydrobaicalin_qt	272.27	HuangQin	GC46	MOL004898	(E)-3-[3,4-dihydroxy-5-(3-methylbut-2-enyl)phenyl]-1-(2,4-dihydroxyphenyl)prop-2-en-1-one	340.40	Gancao
HQ10	MOL002914	Eriodyctiol (flavanone)	288.27	HuangQin	GC47	MOL004903	liquiritin	418.43	Gancao
HQ11	MOL002915	Salvigenin	328.34	HuangQin	GC48	MOL004904	licopyranocoumarin	384.41	Gancao
HQ12	MOL002917	5,2',6'-Trihydroxy-7,8-dimethoxyflavone	330.31	HuangQin	GC49	MOL004905	3,22-Dihydroxy-11-oxo-delta(12)-oleanene-27-alpha-methoxycarbonyl-29-oic acid	512.75	Gancao
HQ13	MOL002925	5,7,2',6'-Tetrahydroxyflavone	286.25	HuangQin	GC50	MOL004907	Glyzaglabrin	298.26	Gancao
HQ14	MOL002926	dihydrooroxylin A	286.30	HuangQin	GC51	MOL004908	Glabridin	324.40	Gancao
HQ15	MOL002927	Skullcapflavone II	374.37	HuangQin	GC52	MOL004910	Glabranin	324.40	Gancao
HQ16	MOL002928	oroxylin a	284.28	HuangQin	GC53	MOL004911	Glabrene	322.38	Gancao
HQ17	MOL002932	Panicolin	314.31	HuangQin	GC54	MOL004912	Glabrone	336.36	Gancao
HQ18	MOL002933	5,7,4'-Trihydroxy-8-methoxyflavone	300.28	HuangQin	GC55	MOL004913	1,3-dihydroxy-9-methoxy-6-benzofurano[3,2-c]chromenone	298.26	Gancao

HQ19	MOL002934	NEOBAICALEIN	374.37	HuangQin	GC56	MOL004914	1,3-dihydroxy-8,9-dimethoxy-6-benzofurano[3,2-c]chromenone	328.29	Gancao
HQ20	MOL002937	DIHYDROOROXYLIN	286.30	HuangQin	GC57	MOL004915	Eurycarpin A	338.38	Gancao
B	MOL000358	beta-sitosterol	414.79	HuangQin	GC58	MOL004917	glycyroside	562.57	Gancao
HQ22	MOL000359	sitosterol	414.79	HuangQin	GC59	MOL004924	(-)-Medicocarpin	432.46	Gancao
HQ23	MOL000525	Norwogonin	270.25	HuangQin	GC60	MOL004935	Sigmoidin-B	356.40	Gancao
HQ24	MOL000552	5,2'-Dihydroxy-6,7,8-trimethoxyflavone	344.34	HuangQin	GC61	MOL004941	(2R)-7-hydroxy-2-(4-hydroxyphenyl)chroman-4-one	256.27	Gancao
HQ25	MOL000073	ent-Epicatechin	290.29	HuangQin	GC62	MOL004945	(2S)-7-hydroxy-2-(4-hydroxyphenyl)-8-(3-methylbut-2-enyl)chroman-4-one	324.40	Gancao
HQ26	MOL000449	Stigmasterol	412.77	HuangQin	GC63	MOL004948	Isoglycyrol	366.39	Gancao
F	MOL001458	coptisine	320.34	HuangQin	GC64	MOL004949	Isolicoflavonol	354.38	Gancao
HQ28	MOL001490	bis[(2S)-2-ethylhexyl] benzene-1,2-dicarboxylate	390.62	HuangQin	GC65	MOL004957	HMO	268.28	Gancao
HQ29	MOL001506	Supraene	410.80	HuangQin	GC66	MOL004959	1-Methoxyphaseollidin	354.43	Gancao
HQ30	MOL002879	Diop	390.62	HuangQin	GC67	MOL004961	Quercetin der.	330.31	Gancao
C	MOL002897	epiberberine	336.39	HuangQin	GC68	MOL004966	3'-Hydroxy-4'-O-Methylglabridin	354.43	Gancao
HQ32	MOL008206	Moslosooflavone	298.31	HuangQin	GC69	MOL000497	licochalcone a	338.43	Gancao
HQ33	MOL010415	11,13-Eicosadienoic acid, methyl ester	322.59	HuangQin	GC70	MOL004974	3'-Methoxyglabridin	354.43	Gancao
HQ34	MOL012245	5,7,4'-trihydroxy-6-methoxyflavanone	302.30	HuangQin	GC71	MOL004978	2-[(3R)-8,8-dimethyl-3,4-dihydro-2H-pyrano[6,5-f]chromen-3-yl]-5-methoxyphenol	338.43	Gancao
HQ35	MOL012246	5,7,4'-trihydroxy-8-methoxyflavanone	302.30	HuangQin	GC72	MOL004980	Inflacoumarin A	322.38	Gancao
HQ36	MOL012266	rivularin	344.34	HuangQin	GC73	MOL004985	icos-5-enoic acid	310.58	Gancao
GC1	MOL001484	Inermine	284.28	Gancao	GC74	MOL004988	Kanzonol F	420.54	Gancao
GC2	MOL001792	DFV	256.27	Gancao	GC75	MOL004989	6-prenylated eriodictyol	356.4	Gancao
GC3	MOL000211	Mairin	456.78	Gancao	GC76	MOL004990	7,2',4'-trihydroxy－5-methoxy-3－	300.28	Gancao

							arylcoumarin		
GC4	MOL002311	Glycyrol	366.39	Gancao	GC77	MOL004991	7-Acetoxy-2-methylisoflavone	294.32	Gancao
GC5	MOL000239	Jaranol	314.31	Gancao	GC78	MOL004993	8-prenylated eriodictyol	356.40	Gancao
GC6	MOL002565	Medicarpin	270.30	Gancao	GC79	MOL004996	gadelaidic acid	310.58	Gancao
GC7	MOL000354	isorhamnetin	316.28	Gancao	GC80	MOL000500	Vestitol	272.32	Gancao
GC8	MOL000359	sitosterol	414.79	Gancao	GC81	MOL005000	Gancaonin G	352.41	Gancao
GC9	MOL003656	Lupiwighteone	338.38	Gancao	GC82	MOL005001	Gancaonin H	420.49	Gancao
GC10	MOL003896	7-Methoxy-2-methyl isoflavone	266.31	Gancao	GC83	MOL005003	Licoagrocarpin	338.43	Gancao
A	MOL000392	formononetin	268.28	Gancao	GC84	MOL005007	Glyasperins M	368.41	Gancao
GC12	MOL000417	Calycosin	284.28	Gancao	GC85	MOL005008	Glycyrrhiza flavonol A	370.38	Gancao
GC13	MOL000422	kaempferol	286.25	Gancao	GC86	MOL005012	Licoagroisoflavone	336.36	Gancao
GC14	MOL004328	naringenin	272.27	Gancao	GC87	MOL005013	18α-hydroxyglycyrrhetic acid	486.76	Gancao
GC15	MOL004805	(2S)-2-[4-hydroxy-3-(3-methylbut-2-enyl)phenyl]-8,8-dimethyl-2,3-dihydropyrano[2,3-f]chromen-4-one	390.51	Gancao	GC88	MOL005016	Odoratin	314.31	Gancao
GC16	MOL004806	euchrenone	406.56	Gancao	GC89	MOL005017	Phaseol	336.36	Gancao
GC17	MOL004808	glyasperin B	370.43	Gancao	GC90	MOL005018	Xambioona	388.49	Gancao
GC18	MOL004810	glyasperin F	354.38	Gancao	GC91	MOL005020	dehydroglyasperins C	340.40	Gancao
GC19	MOL004811	Glyasperin C	356.45	Gancao	D	MOL000098	quercetin	302.25	Gancao
