# Supplementary material for: Ge-Gen-Qin-Lian decoction alleviates the symptoms of type 2 diabetes mellitus with inflammatory bowel disease via regulating the AGE-RAGE pathway
Source: BMC Complement Med Ther. 2024 Jun 10;24:225. doi: 10.1186/s12906-024-04526-x (PMC11163797; doi:10.1186/s12906-024-04526-x)
Supplement: Supplementary file 3 — Additional file 3: File S1: Supplementary methods [file 12906_2024_4526_MOESM3_ESM.pdf]

Supplementary methods

1. **Main reagents**

A high-fat and high-sucrose diet was obtained from Beijing Ke-ao-xie-li Feed Co., Ltd. (Beijing, China). Streptozotocin (STZ) and dextran sulfate sodium salt (DSS) were purchased from Sigma-Aldrich Co., Ltd. (source: St. Louis, MO, USA). Citric acid and sodium citrate were purchased from Sinopharm Chemical Reagent Co., Ltd. (source: Shanghai, China). Methanol, acetonitrile, fomic acid, 2-propanol, and 2-amino-3-(2-chlorophenyl) propanoic acid were purchased from Merck KGaA Co., Ltd. (source: Darmstadt, Germany).

2. **Identification of chemical compounds in GGQLD by UHPLC-MS.**

Step1: Sample preparation

Firstly, 100 mg of GGQLD sample was weighed and placed into a 2 mL centrifuge tube. Secondly, 1mL of 70% methanol with 3 mm steel balls was crushed using an automatic sample rapid grinder (model: JXFSTPRP-48, 70 Hz) for 3 minutes. Subsequently, the mixture was cooled using a low-temperature ultrasound (40 kHz) for 10 minutes. After centrifuging at 12000 rpm for 10 minutes at 4°C, the supernatant was collected and diluted by 2-100 times. Finally, 10 μL of 100 μg/mL internal standard was added and the mixture was filtered through a 0.22 μm PTFE filter for on-board detection.

Step2: Chromatographic separation

The determination was carried out in UHPLC (Thermo Vanquish UHPLC, Thermo Fisher Scientific) and using a C18 column (Zorbax Eclipse C18(1.8μm*2.1mm*100mm), Agilent technologies) with a temperature of 30℃, a flow rate of 0.3 ml/min, a injection volume of 2μL. The mobile phase consisted of phase A, 0.1% fomic acid solution and phase B, pure acetonitrile. A gradient elution was used and the specific conditions of which can be found in following FileS1-table1.

FileS1-table1. The specific conditions of gradient elution program

Time(min)	Flow rate(μL/min)	Gradient	B% Acetonitrile	A% Fomic acid
0-2	300	-	5	95
2-6	300	Linear gradient	30	70
6-7	300	-	30	70
7-12	300	Linear gradient	78	22
12-14	300	-	78	22
14-17	300	Linear gradient	95	5
17-20	300	-	95	5
20-21	300	Linear gradient	5	95
21-25	300	-	5	95

Step3: Mass spectrometry method

The eluents were then analyzed using Mass Spectrometry (Q-Exactive HF, Thermo Fisher Scientific) in both heated electrospray ionization positive and negative modes, of which electrospray boltage was set to 3.5 KV, capillary and heater temperature were separately 330℃ and 325℃, sheath gas flow rate was 45 arb (arbitrary units), aux gas flow was 15 arb, and S-Lens RF Level was 55%. The scanning mode included full scan(m/z 100~1500) and data-dependent mass spectrometry (TopN=5). The resolution of the former is up to 12000, and the latter is up to 60000. The collision mode selected high energy collision dissociation.

Step4: Data processing

The retention time was adjusted, and the peak area was extracted using Compound Discoverer 3.3 software. The chemical components in GGQLD were identified using the Thermo mzCloud online database and the Thermo mzVault local database. We selected 2-Amino-3-(2-chlorophenyl)propanoic acid as an internal label. The concentration of the internal label was set at 1 μg/mL. The relative concentration of chemical components was calculated using the following formula: (internal label concentration/internal label peak area) * chemical components peak area * dilution ratio. The relative percentages were calculated using the following formula: (chemical components peak area / total peak area) * 100%.

3. Primer sequences used in qRT-PCR analyses

The primers for claudin-1, occludin, ZO-1, RAGE, NF-κB, c-JUN, TNF-α, IL-1β and IL-6 were synthesized by Sangon Biotech Technology Co., Ltd., and are listed in FileS1-table2.

FileS1-table2. Specific primers for the qRT-PCR analysis.

Gene	Forward	Reverse
Claudin-1	GCTGGGTTTCATCCTGGCTTCTC	CCTGAGCGGTCACGATGTTGTC
Occludin	TGGCTATGGAGGCGGCTATGG	ACTAAGGAAGCGATGAAGCAGAAGG
ZO-1	AACCCGAAACTGATGCTGTGGATAG	CGCCCTTGGAATGTATGTGGAGAG
TNF-α	CCACGCTCTTCTGTCTACTGA	GATGATCTGAGTGTGAGGGTCT
IL-6	AGCCACTGCCTTCCCTAC	TTGCCATTGCACAACTCTT
IL-1β	TTCAAATCTCACAGCAGCAT	CACGGGCAAGACATAGGTAG
RAGE	AGAAACCGGTGATGAAGGACA	GGTTGTCGTTTTCGCCACAG
c-JUN	CAAACCTCAGCAACTTCAACC	CTGGGACTCCATGTCGATG
β-actin	ACGGTCAGGTCATCACTATCG	GTTTCATGGATGCCACAGGATT
